# *CircACVR2A*(2–4) regulates stem cell pluripotency by uncoupling CDK-activating kinase functions

**DOI:** 10.1093/nar/gkag964

**Published:** 2026-10-13

**Authors:** Stuart Webb, Vanessa M Conn, Eljo Yvette van Battum, Kirsty Kirk, Ashleigh B Geiger, Suman Singh, Marta Gabryelska, Michael J Roach, Shashikanth Marri, He Lin, Ryan Liu, Jordyn Binelli, John Toubia, Jenne Tran, Alex D Colella, Thomas Colunga, Michael Kulik, Cedric Bardy, Lachlan Jolly, R Jeroen Pasterkamp, Ross A McKinnon, Stephen Dalton, Lasse S Kristensen, Adrienne E Sullivan, Simon J Conn

**Affiliations:** Flinders Health and Medical Research Institute, College of Medicine & Public Health, Flinders University, Bedford Park, SA 5042, Australia; Department of Genetics and Molecular Pathology, Centre for Cancer Biology, an alliance between SA Pathology and the University of South Australia, Adelaide, SA 5000, Australia; Flinders Health and Medical Research Institute, College of Medicine & Public Health, Flinders University, Bedford Park, SA 5042, Australia; Department of Translational Neuroscience, University Medical Center Utrecht Brain Center, Utrecht University, Universiteitsweg 100, 3584 CG Utrecht, The Netherlands; Flinders Health and Medical Research Institute, College of Medicine & Public Health, Flinders University, Bedford Park, SA 5042, Australia; Adelaide Centre for Epigenetics, School of Biomedicine, Faculty of Health and Medical Sciences, Adelaide University, Adelaide 5000, Australia; South Australian immunoGENomics Cancer Institute, Faculty of Health and Medical Sciences, Adelaide University, Adelaide 5000, Australia; Flinders Health and Medical Research Institute, College of Medicine & Public Health, Flinders University, Bedford Park, SA 5042, Australia; Flinders Health and Medical Research Institute, College of Medicine & Public Health, Flinders University, Bedford Park, SA 5042, Australia; Flinders Health and Medical Research Institute, College of Medicine & Public Health, Flinders University, Bedford Park, SA 5042, Australia; Flinders Health and Medical Research Institute, College of Medicine & Public Health, Flinders University, Bedford Park, SA 5042, Australia; Flinders Health and Medical Research Institute, College of Medicine & Public Health, Flinders University, Bedford Park, SA 5042, Australia; Flinders Health and Medical Research Institute, College of Medicine & Public Health, Flinders University, Bedford Park, SA 5042, Australia; Flinders Health and Medical Research Institute, College of Medicine & Public Health, Flinders University, Bedford Park, SA 5042, Australia; Centre for Cancer Biology, an alliance between SA Pathology and the University of South Australia, Adelaide, SA 5000, Australia; ACRF Cancer Genomics Facility, SA Pathology, Adelaide, SA 5000, Australia; Flinders Health and Medical Research Institute, College of Medicine & Public Health, Flinders University, Bedford Park, SA 5042, Australia; Lifelong Health, South Australian Health and Medical Research Institute, Adelaide, South Australia 5000, Australia; Flinders Health and Medical Research Institute, College of Medicine & Public Health, Flinders University, Bedford Park, SA 5042, Australia; Center for Molecular Medicine, Department of Biochemistry and Molecular Biology, University of Georgia, Athens, GA 30602, United States; Center for Molecular Medicine, Department of Biochemistry and Molecular Biology, University of Georgia, Athens, GA 30602, United States; Flinders Health and Medical Research Institute, College of Medicine & Public Health, Flinders University, Bedford Park, SA 5042, Australia; Lifelong Health, South Australian Health and Medical Research Institute, Adelaide, South Australia 5000, Australia; School of Pharmacy and Biomedicine, Adelaide University, Adelaide, SA 5000, Australia; Robinson Research Institute, Adelaide University, Adelaide, SA 5000, Australia; Department of Translational Neuroscience, University Medical Center Utrecht Brain Center, Utrecht University, Universiteitsweg 100, 3584 CG Utrecht, The Netherlands; Flinders Health and Medical Research Institute, College of Medicine & Public Health, Flinders University, Bedford Park, SA 5042, Australia; School of Biomedical Sciences, The Chinese University of Hong Kong, Hong Kong,China; Department of Biomedicine, Aarhus University, Aarhus 8000, Denmark; Adelaide Centre for Epigenetics, School of Biomedicine, Faculty of Health and Medical Sciences, Adelaide University, Adelaide 5000, Australia; South Australian immunoGENomics Cancer Institute, Faculty of Health and Medical Sciences, Adelaide University, Adelaide 5000, Australia; Flinders Health and Medical Research Institute, College of Medicine & Public Health, Flinders University, Bedford Park, SA 5042, Australia

## Abstract

Differentiation of stem cells is driven by tightly coordinated transcriptional programs, yet the contribution of circular RNAs (circRNAs) to lineage specification remains poorly defined. Here, we profiled circRNA and messenger RNA expression during differentiation of human embryonic stem cells into five germ layer–derived lineages. Targeted silencing of dynamically regulated circRNAs revealed that depletion of *circACVR2A*(2–4), but not its linear counterpart, induced rapid loss of pluripotency accompanied by neuroectoderm-biased differentiation, phenotypically and transcriptionally. Mechanistically, *circACVR2A*(2–4) interacts with cyclin H within the cyclin-dependent kinase (CDK)-activating kinase (CAK) complex to modulate RNA polymerase II (RNAPII) phosphorylation independently of CDK activation, with lineage commitment consistent with destabilization of RNAPII pausing at poised neuroectodermal loci. *In vivo, circACVR2A*(2–4) silencing impaired neuronal migration in the embryonic mouse brain and attenuated glioblastoma progression in mouse orthotopic xenografts. Together, these findings identify a circRNA-mediated mechanism that uncouples CAK complex activity to modulate stem cell fate.

## Graphical Abstract

**Graphical Abstract:**
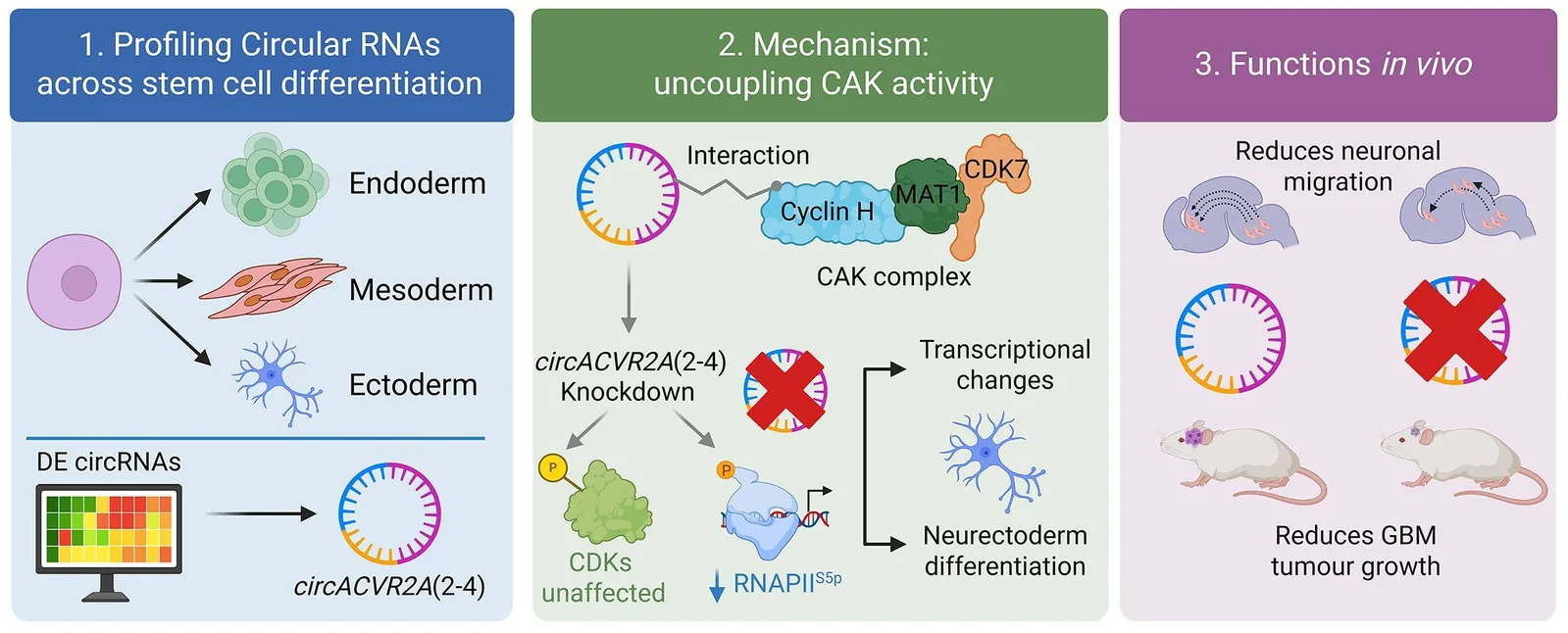

## Introduction

Stem cell differentiation is a widely used model for examining human cellular development and disease processes [1]. Human embryonic stem cells (hESCs) are a physiologically relevant cell system as they have the capacity to differentiate into any cell of the body originating via the three germ layers—ectoderm, mesoderm, and endoderm [2]. Importantly, stem-like cell states are also a defining feature of cancers such as glioblastoma (GBM), where glioma stem cells (GSC) drive disease aggressiveness, therapeutic resistance, and recurrence, highlighting the physiological and translational relevance of stem cell biology [3]. Transcriptomic profiling of coding and non-coding RNA in stem cells and their differentiated progeny can, therefore, illuminate cell- and disease-specific RNA molecules [4].

Circular RNAs (circRNAs) constitute a class of covalently closed, largely non-coding RNAs generated via backsplicing of pre-messenger RNA (mRNA) (Fig. 1A) or non-coding RNA transcripts. CircRNAs primarily comprise canonical coding exons; however, exonic–intronic, intronic, or intergenic circRNAs are readily detectable with RNA-sequencing and bioinformatics pipelines [5–7]. While expressed across all kingdoms of life [8], circRNAs are not pervasive and have been shown to be cell type-specific [9]. Circularization of these transcripts can be driven by complementary sequences, including *Alu* repeats, flanking the circRNA exons, or regulated by several *trans*-acting RNA-binding proteins and splicing factors [10]. CircRNAs have been shown to interact with proteins, RNA [microRNA (miRNA), mRNA, and snRNA], and DNA, as well as encoding for proteins, although translation of circRNAs is a rare phenomenon for a small number of circRNAs and requires an internal ribosome entry site (IRES)-like motif [7]. In addition, several circRNAs have been found to directly affect the regulation of their parental genes by impacting transcription [11], protein complex stability [12], gene methylation [13], and splicing [14, 15].

While an extensive number of reports have detailed the mRNA expression changes occurring during the differentiation of hESCs along various lineages, those describing changes in circRNA expression are limited to single-lineage differentiation paradigms [16, 17]. Therefore, to provide insight into coordinated or cell-type specific circRNA regulation and reveal circRNA candidates for further functional studies, this study employed simultaneous profiling of circRNAs and mRNAs during hESC differentiation into five lineages by RNA sequencing (RNAseq). This was followed by targeted screening of candidate circRNAs by short hairpin RNA (shRNA) silencing and phenotypic analysis, followed by comprehensive *in vitro* and *in vivo* functional analysis of the circRNA eliciting the strongest phenotype, *circACVR2A*(2–4), in embryonic neurons and GBM.

## Materials and methods

### Cell lines

All human cell lines were incubated at 37°C with 5% CO_2_. Human embryonic kidney cells (HEK293T, ATCC® CRL-3216™), human lung adenocarcinoma cells (A549, ATCC® CCL-185™), and mouse fibroblast cells (NIH/3T3 cells, ATCC® CRL-1658™) were grown in Dulbecco’s modified Eagle’s medium (GIBCO BRL) with 10% foetal bovine serum (Thermo Fisher Scientific). H9 hESCs (WA09, WiCell®, WAe009-A) and H1 hESCs (WA01, WiCell®, WAe001-A) were grown in Gibco™ StemFlex™ Medium and cultured on Gibco™ Vitronectin recombinant human protein, truncated (VTN-N, Thermo Fisher Scientific, Cat. #A14700) or GelTrex™-coated (Thermo Fisher Scientific, Cat. #A1413202) dishes, or in mTeSR+ (STEMCELL Technologies) on GFR Matrigel-coated plates. H9 hESCs were authenticated by short tandem repeat profiling and confirmed to lack structural and/or numerical abnormalities of chromosomes by karyotyping.

### Generation of FUCCI H9 hESC cell line

To generate the H9 hESC cell line with stable expression of tFucci (CA)5, hyperactive PiggyBac transposase expression vector (pPB-CMV-HyPBase) and tFucci (CA)5 (Addgene, cat. no.153521, kindly provided by Atsushi Miyawaki) were co-transfected into fully dissociated H9 hESCs using Fugene 4K (Promega) according to manufacturer’s instructions. Fucci (CA)-expressing single cells were selected by fluorescence-activated cell sorting.

### Short hairpin RNA and circRNA overexpression constructs

All human RNA-targeting shRNAs were cloned into pLKO.1-puro (Addgene, #8453), which was sequentially digested with *Eco*RI-HF® and *Age*I-HF® (New England Biolabs, Ipswich, MA, USA) in 1× CutSmart buffer at 37°C for 1 h and gel-purified. Oligonucleotides designed to target specific candidate RNAs (circRNAs and mRNAs) were annealed to generate complementary overhangs, which were ligated into pLKO.1-puro with T4 DNA ligase (New England Biolabs) (Supplementary Table S4). For targeting circRNAs, 21-mer shRNAs were designed so that the BSJ was between nucleotides 10 and 11, where possible, with additional shRNAs moved 1–4 nt in the 5′ or 3′ direction. For the silencing of mouse circRNAs, the oligonucleotides were cloned into the *Hpa*I/*Xho*I sites of PLL3.7 vector, which also expressed eGFP as a marker of cell transfection under the control of CMV promoter [18]. Knockdown efficiency of these constructs was confirmed in NIH/3T3 cells, which expresses *mmu_circAcvr2a*(2–4), by RT-qPCR. Overexpression of circRNAs was achieved as per Liu *et al*. [19], using circR plasmid for transient overexpression in HEK293T cells and CircRNA Mini vector (AddGene #60648) for H9 hESCs by insertion of *ACVR2A* exons 2–4 into the *Pac*I/*Fse*I and *Sac*II*/Cla*I restriction sites, respectively. Constructs were Sanger-sequenced using T7 promoter and/or BGHA reverse primers. For making stable overexpression lines of *circACVR2A*(2–4) in CircRNA Mini vector, plasmids were transfected into H9 hESCs using Lipofectamine 2000. After 24 h, selection (50 µg/ml G418) was applied for 4–7 days. RT-PCR amplicons for *circACVR2A*(2–4) from EV and overexpression lines were gel-purified and underwent Sanger sequencing.

### Lentiviral transduction of shRNA constructs

Lentiviral particles were generated in HEK293T cells with psPAX2 packaging plasmid and VSVg envelope plasmid. Target cells (hESCs) were exposed to virus-containing supernatant and selected with 3 µg/ml puromycin (P8833, Merck) for 3 days, starting 24 h after transduction.

### Circular RNA sequencing

RNA was treated with DNase I (Roche) at 37°C for 15 min and purified with the RNeasy RNA MinElute Cleanup Kit (Qiagen, Venlo, Netherlands). Two synthetic circRNA spike-ins were added to the purified RNA at 10-fold difference to permit normalization across samples [20]. RNA (2.5 µg) was digested with five units of RNase R (2 U/μg RNA), then purified with RNeasy Micro Kit (Qiagen, Venlo, Netherlands). RNA was then depleted for ribosomal RNA (rRNA) using the NEBNext® rRNA Depletion Kit (Human/Mouse/Rat) (New England Biolabs, Ipswich, MA, USA) before libraries were constructed with the KAPA RNA HyperPrep Kit for illumina® (KAPA Biosystems). RNase R-treated RNA-seq libraries from three biological replicates of pluripotent stem cells (H9) and five differentiated states (cardiomyocytes, definitive endoderm, dopaminergic neurons, floor plate precursors, and hepatocytes) were multiplexed and sequenced on the Illumina NextSeq 500 platform using the stranded, single-end protocol with a read length of 150. Raw reads were adapter-trimmed and filtered for short sequences using cutadapt v1.8.1 [21], setting the minimum-length option to 18, error-rate 0.2, quality cut-off 28, and overlap 5. The resulting FASTQ files, averaging 35 million reads per sample, were analysed and quality-checked using the FastQC program (http://www.bioinformatics.babraham.ac.uk/projects/fastqc). Reads were mapped against the human reference genome (hg19) using the STAR spliced alignment algorithm [22] (version 2.5.3a with default parameters and –chimSegmentMin 20, –quantMode GeneCounts), returning an average unique alignment rate of 54%. Abundance estimations of circRNAs were conducted utilizing the chimeric junction file produced by the STAR aligner as processed by the CIRCexplorer2 (version 2.3.2 using default parameters) annotate function. Differential expression analysis was evaluated from TMM-normalized circRNA counts using R (version 3.2.3) and edgeR [23] (version 3.3) following protocols as described [24]. Alignments were visualized and interrogated using the Integrative Genomics Viewer v2.3.80 [25].

### Messenger RNA sequencing

Libraries were prepared using the NEBNext Ultra II Directional RNA-Library Prep Kit for Illumina (New England Biolabs, Ipswich, MA, USA). PolyA mRNA was isolated using the Poly(A) mRNA Magnetic Isolation Module (NEB#E7490) (New England Biolabs, Ipswich, MA, USA) using the same DNase I treated RNA from the circ-RNA seq library prep. Prepared libraries were sequenced at GENEWIZ (Suzhou, China) using Illumina HiSeq 4000 Platform using the PE150 protocol. Raw reads were trimmed and filtered for short sequences using cutadapt v.1.9.1 [21], setting the minimum-length option to 18, error-rate 0.2, and overlap 5. The resulting FASTQ files, averaging 39 million reads per sample, were analysed and quality-checked using FastQC program (http://www.bioinformatics.babraham.ac.uk/projects/fastqc). Reads were mapped against the human reference genome (hg38) using STAR spliced alignment algorithm [22] (version 2.7.0 with default parameters), returning an average of unique alignment rate of 82%. Using featureCounts [26], reads were assigned to each exonic region to derive counts, and differential expression analysis was evaluated from filtered RPKM-normalized counts using DESeq2 statistical tool [27]. Alignments were visualized and interrogated using the Integrative Genomics Viewer v2.3.80 [25].

### Total RNA-seq

Libraries were prepared using the TruSeq Stranded Total Library Prep Kit for Illumina. Prepared libraries were sequenced at GENEWIZ (Suzhou, China) using HiSeq 2500 (Illumina) platform using the paired-end protocol with read length of 150. Raw reads were trimmed and filtered for short sequences using cutadapt v.1.9.1 [21], setting minimum-length option to 18, error-rate 0.2, and overlap 5. The resulting FASTQ files, averaging 124 million reads per sample, were analysed and quality-checked using FastQC program (http://www.bioinformatics.babraham.ac.uk/projects/fastqc). Reads were mapped against the human reference genome (hg38) using STAR spliced alignment algorithm [22] (version 2.7.0 with default parameters and –chimSegmentMin 20, –quantMode GeneCounts), returning an average of unique alignment rate of 88%. Differential expression analysis was evaluated from filtered RPKM-normalized gene counts using DESeq2 statistical tool [27]. Abundance estimations of circRNAs were conducted utilizing the chimeric junction file produced by the STAR aligner as processed by the CIRCexplorer2 (version 2.3.3 using default parameters) annotate function [28]. Alignments were visualized and interrogated using the Integrative Genomics Viewer v2.3.80 [25].

### Real-time, quantitative reverse transcriptase PCR

RT-qPCR was performed according to the MIQE 2.0 guidelines[29]. For RT-qPCR, RNA was reverse transcribed with QuantiTect® reverse transcription kit (Qiagen, Venlo, Netherlands). For most targets, QuantiTect® SYBR® Green PCR kit was used in a 10 µl reaction volume on RotorGene Q (Qiagen, Venlo, Netherlands). Reactions were performed in technical and biological triplicates, including no-RT and no-template controls. Absolute quantitation was performed for targets with purified and quantified amplicons across seven orders of magnitude (10^1^–10^7^ copies per reaction) to generate a standard curve. RNA from a known cellular input numbers was utilized in complementary DNA (cDNA) synthesis, and a comparison of the Ct values from these cells against the standard curves was used to determine copy numbers per cell.

### 3-Dimensional RNA fluorescence *in situ* hybridization

3-Dimensional RNA fluorescence *in situ* hybridization (3D RNA FISH) was performed as per Conn *et al*. [30] on H9 hESCs cells grown on vitronectin-coated glass coverslips Localization of *circACVR2A*(2–4) was achieved using an *in vitro* transcribed RNA probes, sense (negative control) and antisense (circRNA targeting), to the back-splice junction and labelled with Alexa Fluor 488 using the FISH Tag RNA Multicolor kit (Thermo Fisher Scientific) (Supplementary Table S4). Nuclei were counterstained with DAPI in Vectashield mounting medium. Slides were imaged using the Zeiss LSM880 confocal microscope (Zeiss, Oberkochen, Germany) and *z*-stacks obtained using optimized intervals (z-voxel ~0.41 mm), 40× objective and 4.63× zoom.

### Neuronal differentiation of induced pluripotent stem cells

Differentiation of induced pluripotent stem cells into neurons (day 30 and day 60 timepoints) was performed as per Stringer *et al*. [31] in BrainPhys medium (STEMCELL Technologies). RT-qPCR was performed using TaqMan assays (Thermo Fisher Scientific)(Supplementary Table S4) in TaqMan Fast Advanced Master Mix (Thermo Fisher Scientific).

### Gene Ontology enrichment analysis

Gene Ontology enrichment analysis of gene sets from RNA-seq data was performed using the Gene Ontology Resource [32]. The tool was run by inputting gene names from RNA-seq data at defined cutoff thresholds, selecting biological process and *Homo sapiens* as the species.

### RNA immunoprecipitation-mass spectrometry

Biotinylated (5′TEG-biotin), 30-nt-long DNA oligonucleotides were designed to span the BSJ of *circACVR2A*(2–4) (Integrated DNA Technologies)—antisense strand 5′-TAC CAA GTA TAG CAC TTG AGT TGG AAC AAG-3′, and sense strand (negative control) 5′-CTT GTT CCA ACT CAA GTG CTA TAC TTG GTA -3′. BLAST analysis of probes revealed no significant matches and secondary structure folding assessment with RNAfold showed no strong secondary structure. CircRNA pulldown was performed as per Gabryelska *et al*. [33] using unfixed HEK293T cells, transiently transfected (40 h) with pcDNA3.1::*circACVR2A*(2–4). Three biological replicates were prepared for both the antisense and sense (control) probes.

Protein eluate from the circRNA pulldown was concentrated, and protein was quantified with Bio-Rad Protein Assay Dye reagent concentrate. One microgram of total protein from each sample was reduced by the addition of dithiothreitol (Astral Scientific) to a concentration of 10 mM and incubated for 30 min at 56°C. The samples were alkylated by the addition of 2-chloroacetamide (Merck) to a concentration of 20 mM and incubated at room temperature in the dark from 30 min. The samples were then digested with trypsin (1:50 enzyme-to-substrate ratio) (Promega) and incubated overnight at 37°C. Peptides were analysed with a Dionex Ultimate 3000 UPLC coupled with an Orbitrap Exploris 480 tandem mass spectrometer (Thermo Fisher Scientific, Waltham, Massachusetts, USA). An inhouse analytical column created from 75-µm inner diameter fused silica capillary with an integrated pulled tip emitter, packed with 1.9-µm ReproSil-Pur C18 beads (Dr Maisch, Ammerbuch, Germany) to 25 cm, coupled with an inhouse-packed trap column made from 300-µm inner diameter fused silica capillary, packed with 1.9-µm ReproSil-Pur C18 beads to 5 mm, was used. Mobile phase A was 0.1% formic acid in water, and mobile phase B was 0.1% formic acid in 80% acetonitrile. For each injection, 1 µg of peptides was loaded and separated using a 60-min gradient from 3% to 50% mobile phase B, followed by a 35-min washing and equilibration gradient. Samples were analysed using data-dependent acquisition (DDA) utilizing a top-20 dependent scan instrument method. Briefly, ms1 scans were performed using an Orbitrap resolution of 60 000 and a scan range from 350 to 1200 m/z. A normalized AGC target of 3e6 with auto maximum injection time mode used. An intensity threshold of 5e4 and dynamic exclusion time of 20 s were employed for all data-dependent ms2 scans that were acquired at 15 000 resolution, AGC target 7.5e4, and 30% normalized collision energy in the HCD cell, with auto maximum injection time mode used.

DDA data files were processed using Proteome Discoverer 2.4.0.305 software (Thermo Fisher). MS/MS spectra were searched against the *Homo sapiens* reference proteome database (SwissProt v2017-10-25) appended with a common database of laboratory contaminants. The processing workflow utilized the Sequest HT search engine. Precursor mass tolerance was set to 10 ppm, and fragment mass tolerance was set to 0.02 Da. The enzyme specificity was set to trypsin, allowing for a maximum of two missed cleavages. Carbamidomethylation of cysteine residues was defined as a static modification. Dynamic modifications included oxidation of methionine, and deamidation of asparagine and glutamine, and Gln→pyro-Glu of the protein N-terminus. Peptide-spectrum matches were validated using the Percolator node, applying a target-decoy search strategy. False discovery rates (FDRs) were strictly filtered to a 1% threshold at both the peptide and protein levels. For label-free quantification, precursor ion intensities were extracted using the Minora Feature Detector node. Protein hits were identified by the following filtering criteria: (i) rejecting proteins with only a single peptide, (ii) having to be present in all biological replicates for the antisense (positive) strand pulldowns, and (iii) absent from all three sense (negative control) pulldowns.

### RNA immunoprecipitation

Forty hours post-transfection of pcDNA3.1::CCNH-FLAG and circR::*circACVR2A*(2–4), cells were washed with ice-cold phosphate-buffered saline (PBS) and, for CLIP samples only, UV-irradiated (254 nm, 0.15 J/cm^2^) with a Stratalinker while plates were left on ice. Three million HEK293T cells were collected by scraping and centrifuged at 4°C at 1000 × *g* for 5 min. Cells were washed twice with 1× ice-cold PBS before being resuspended in 1 ml of RIPA buffer [150 mM NaCl, 1% (v/v) Nonidet *P*-40, 0.5% sodium deoxycholate, 0.1% sodium dodecyl sulphate (SDS), 50 mM Tris (pH 7.4), 200 units/ml RNase OUT, 1× cOmplete Tablets, Mini ethylenediaminetetraacetic acid-free (Roche, Basel, Switzerland), 1 mM sodium orthovanadate, and 1 mM sodium fluoride). The lysate was rotated at 4°C for 30 min. Input samples were collected for protein (100 μl) and RNA (50 μl) purification, with the remaining lysate split into two 500-μl samples—one for control IgG IP and one for FLAG IP. Dynabeads™ magnetic beads (Thermo Fisher, Waltham, Massachusetts) were rotated for 5 min to resuspend the beads. Thirty microliters of Dynabeads™ magnetic beads were added to a 1.5-ml tube. The magnet was applied to separate the beads from the solution, and the supernatant was discarded. Antibodies [5 μg anti-FLAG and Mouse IgG (sc-2025, Santa Cruz)] were diluted in 200 μl PBS with Tween™ 20 and added to the tube containing the Dynabeads™ magnetic beads. The bead-antibody mixture was incubated for 20 min at room temperature. The bead-antibody mixture was placed in the magnetic stand, and the supernatant was removed, leaving the antibody-conjugated beads. The conjugated beads were washed with 200 μl of PBS with 0.1% Tween™ 20, and the mixture was washed by gentle pipetting. The antibody-conjugated beads tube was put on the magnet to remove the supernatant, and the prepared lysate (500 μl) was added directly to the beads. The sample was incubated for 4 h at 4°C with rotation to allow beads to bind to the antigen. The tube was placed on the magnet, and the supernatant was removed. The antigen-antibody-bead complex was washed three times by gentle pipetting, adding 200 μl of RIPA buffer, then placing the tube on the magnet, and removing the supernatant. After the last wash, the beads were separated into two tubes for RNA and protein. For RNA, 25% of the beads were resuspended in 500 μl TRIzol, and RNA was purified with the Direct-zol^TM^ RNA MiniPrep Kit (Zymo Research). For protein, the antigen was eluted from the beads by boiling 75% of the beads in 1× SDS loading dye [50 mM Tris–HCl, pH 6.8, 6% (w/v) glycerol, 2% SDS, 0.004% bromophenol blue, and 1% 2-mercaptoethanol) at 95°C for 5 min.

### Western blotting

Western blotting was performed as per Conn *et al*. [34] using the following antibodies, primary: anti-FLAG M2 mouse monoclonal (F1804, Sigma–Aldrich), cyclin H rabbit polyclonal (#2927, Cell Signaling Technology), phospho-cyclin-dependent kinase (CDK) substrate rabbit monoclonal [pTPXK] (#14371, Cell Signaling Technology), ACVR2A mouse monoclonal (sc-515826, Santa Cruz Biotechnology), RNA polymerase II (RNAPII) mouse monoclonal antibody (C15200253, Diagenode), and RNAPII S5p mouse monoclonal (C15200007-50, Diagenode); and secondary: goat anti-mouse HRP (Cat #31430, Thermo Fisher Scientific) and goat anti-rabbit HRP (Cat #31460, Thermo Fisher Scientific). SuperSignal™ West Pico PLUS chemiluminescent substrate (Cat #34577, Thermo Fisher Scientific) was used to develop the HRP signal, which was imaged using the ChemiDoc XRS+ (Bio-Rad).

### CAK assay

This assay was performed as per the manufacturer’s instructions (CDK7/CyclinH1/MNAT1 Kinase Enzyme System with ADP-Glo, Promega Cat #VA7402). *CircACVR2A*(2–4) and *circMLL*(9,10) were *in vitro* transcribed, ligated, and purified as per Conn *et al*. [20]. These were added into the reaction mixture at 1:1000, 1:100, and 1:10 molar ratio with the 30 ng of CDK-activating kinase (CAK) complex and read on the ClarioSTAR luminometer (BMG Labtech) in white, 96-well trays. The standard reagent included with the kit was myelin basic protein (MBP), and an additional CDK7/9tide peptide substrate (YSPTSPSYSPTSPSYSPTSPSKKKK, Thermo Fisher Catalogue #PV5090) was utilized.

### AlphaLISA™ SureFire® Ultra™ assays

Quantification of total and phosphorylated CDK proteins in ES cell lysates was performed using AlphaLISA™ SureFire® Ultra™ assays (Revvity) as per manufacturer’s instructions. Assays used were for total CDK1 (#ALSU-TCDK-A 500, LOT #U10821); p-CDK1 Thr14 (#ALSU-PCDK1-A 500, LOT #U10816); p-CDK1 Thr161 (#ALSU-PCDK1-A 500, LOT #U10815); total CDK2 (#ALSU-TCDK2-A 500; LOT #U10818), p-CDK2 Thr160 (#ALSU-PCDK1-A 500, LOT #U10817); total CDK4 (#ALSU-TCDK4-A 500; LOT #U10819); and total CDK7 (#ALSU-TCDK7-A 500; LOT #U10820). White, 384-well plates were scanned using the EnSight™ multimode detector (PerkinElmer).

### Immunofluorescence

Cells were washed with PBS and fixed with 4% formaldehyde for 15 min at room temperature, then washed (PBS) and permeabilized with permeabilization buffer (0.3% Triton X-100 in PBS) for 5 min at room temperature. Cells were then washed (PBS) and blocked with blocking buffer [10% foetal bovine serum (v/v), 3% bovine serum albumin (BSA) (w/v) in PBS] at room temperature for 1 h prior to incubation with primary antibodies at 4°C overnight in primary antibody dilution buffer (1% BSA and 0.3% Tween in PBS). Following overnight incubation with primary antibodies, cells were washed (0.3% Tween in PBS) and incubated with secondary antibodies in blocking buffer for 1 h at room temperature. Cells were washed again, and nuclei were stained using DAPI. Primary antibodies were LHX5 (RDSAF6290, R&D Technologies), Pol II (C15200004, Diagenode), and Pol II S5p (C15200007, Diagenode). Secondary antibodies used were goat anti-mouse Alexa Fluor® 488 (A11001, Thermo Fisher Scientific) and goat anti-rabbit Alexa Fluor® 488 (A11008, Thermo Fisher). Imaging was performed with a Zeiss AxioObserver microscope. Images were quantified with Fiji using the StarDist nuclear segmentation plugin [35], and non-nuclear objects were excluded based on size and circularity in R. All plotting was performed using R package ggplot2.

### RNA polymerase II inhibitor screen

H9 hESCs were seeded as small aggregates in mTeSR+ in a 96-well Matrigel-coated plate. Twenty-four hours after seeding, media were refreshed and inhibitors diluted in dimethyl sulfoxide (DMSO) were added. Media and inhibitors were refreshed daily. After 48 h of inhibitor treatment, plates were fixed for analysis by immunofluorescence against LHX5 with DAPI staining. Inhibitors and final concentrations used were KB-0742 dihydrochloride (SEL-S9901, 1 µM), samuraciclib (ICEC0942) hydrochloride (SEL-S8722, 60 nM), LCD4297 (SEL-S7992, 200 nM), and MS417 (SEL-S7615, 125 nM).

### siRNA knockdown of *circACVR2A*(2–4)

Non-targeting (NT) siRNA (GenePharma) and two custom siRNAs targeting *circACVR2A*(2–4) (si1: UCAAGUGCUAUACUUGGUATT; si2: CAACUCAAGUGCUAUACUUTT) were resuspended in DNase/RNase-free distilled water to a stock concentration of 20 µM. H9 hESCs were transfected with siRNAs with Lipofectamine RNAiMAX (Thermo Fisher) according to manufacturer’s reverse transfection protocol. Briefly, siRNAs (final concentration 2 or 5 nM) were incubated with Lipofectamine RNAiMAX reagent in Opti-MEM reduced serum media (Thermo Fisher) for 20–30 min at room temperature. H9 hESCs were dissociated to single cells and suspended in mTeSR+ with Y-27632 (STEMCELL Technologies #72308, final concentration 10 µM). Cells were added to wells and gently swirled to distribute, then incubated at 37°C. Sixteen hours after transfection, media were refreshed to remove Y-27632. Media were refreshed daily until cells were collected for RNA extraction and/or immunofluorescence at 48 and 72 h post-transfection.

### ChIP-Seq analysis

ChIP-Seq data for H1 hESCs cells were accessed from GEO for Total RNAPol2 (GSM1579359) and RNAPol2 Ser5p (GSM1579351) [36]. Lists of the top 100 genes specific to pluripotent, mesoderm, endoderm, and ectoderm cell types were generated by performing differential gene analysis in Loupe between scRNA-Seq clusters of BMP4-differentiated hESCs (GSE301542) [37]. Heatmaps showing ChIP-Seq signal within a 4 kb window, centred at the transcription start site (TSS) for these genes were generated using deepTools.

### Digital PCR

Digital PCR was performed to quantify *mmu_circAcvr2a*(2–4) abundance normalized to *GAPDH* expression. The QIAcuity EG PCR Kit (Qiagen, Cat. #250111) was used, comprising 3× EvaGreen PCR Master Mix, 0.4 μM of each forward and reverse primer, and RNase-free water to the final reaction volume of 35 µl for 26k Qiacuity 26k Nanoplate (Qiagen, Cat. #250001). The reaction mix was first dispensed into the standard PCR tube, followed by the addition of 5 µl template cDNA, with the same volume of nuclease-free water used for the non-template control. The contents of each tube were thoroughly mixed, then transferred to the nanoplate and sealed with the nanoplate seal. The cycling conditions include initial activation at 95 °C for 2 min, followed by 40 cycles of 95 °C for 15 s, 55°C for 15 s, and 72 °C for 15, with a final 5 min cooling step, with imaging performed on the QIAcuity One, 5plex System (Qiagen, Cat. #911022), green channel with a 200 ms exposure.

### *In utero* electroporation

All animal use and care were in accordance with institutional guidelines and approved by the Animal Ethics Committee of Utrecht University (CCD license AVD115002016532) and conducted in agreement with Dutch laws (Wet op de Dierproeven, 1996, revised 2014) and European regulations. C57Bl/6j (Charles River Laboratories IMSR_JAX:000 664) were bred in-house. Animals were socially housed with sawdust bedding and tissues under a 12 h light–dark cycle. The morning of a vaginal plug was considered embryonic stage (E)0.5.

*In utero* electroporation was performed as described [38]. Pregnant female mice at embryonic stage (E)14 were deeply anesthetized in 5% isoflurane in O_2_ and injected with 0.05 mg/kg buprenorphin (Temgesic) in physiological salt solution subcutaneously. Anaesthesia was maintained at 2% isoflurane in O_2_. A patch of abdominal hair was removed using Veet® crème before skin and muscles of the abdominal wall were opened and the uterus containing embryos was exposed. Embryos were kept wet with warm PBS during the procedure. Using a calibrated pulled glass needle (borosilicate glass, 1 mm OD, 0.58 mm ID, World Precision Instruments) and a picospritzer (PLI-100, Harvard Apparatus), embryos were injected with 1.2 μl of DNA mix [1 μg/μl plasmid DNA in 0.1% FastGreen (Sigma) in MQ] in lambda to fill both lateral ventricles. Scrambled control and short-hairpin conditions were spread among embryos within each mother to standardize variation between mothers and reduce animal use. DNA was targeted to the motor cortex using five electrical pulses of 35 mV (50 ms duration at 950 ms interval) (BTX 830 square wave electroporation system) applied with triple electrodes [39]: 10 mm platinum tweezer electrodes (Harvard Apparatus) were coupled to the negative pole and a 3 mm gold-plated peddle electrode (Harvard Apparatus) was coupled to the positive pole. The uterus was carefully placed back, and muscles and skin were sutured separately. Post-surgical pain was relieved with 0.027 mg/ml carpofen (Carporal) in drinking water and wet food. The mother mouse was killed by cervical dislocation at E17. Embryo brains were isolated and fixed in ice-cold 4% paraformaldehyde in PBS ON, incubated in 30% sucrose in PBS ON, embedded in 7.5% gelatin/10% sucrose in PBS, and frozen at −35°C. Twenty-micrometre cryostat sections were blocked in 2% gelatin/0.25% Triton X-100 in PBS (PBS-GT) and incubated with 1:1000 chicken-anti-GFP (AVES) in PBS-GT ON at RT. After PBS washes, sections were incubated with 1:750 donkey anti-chicken IgY-Alexa 488 (Jackson ImmunoResearch) in PBS-GT for 2 h at RT and counterstained with DAPI. Slides were mounted with Mowiol. Images were taken of three sections per animal using an epifluorescent microscope (Axioskop, Zeiss) at 10× magnification. In Photoshop (Adobe CC 2024), three 6-square bins were placed on each image using the DAPI signal, so that bin 1–2 covered the intermediate zone and bin 4–6 covered the cortical plate (Fig. 6F). All GFP-positive migrating neurons inside the bins were manually counted. The percentage of cells in each bin was averaged from three 6-square bins per section, from three sections per brain showing the corpus collosum. Data were averaged from *N* = 9 embryos from 6 mothers (Scrambled), and 7 embryos from 3 mothers (*mmu_circAcvr2a*(2–4) shRNA2). The average percentage of cells per bin was compared with multiple non-parametric Mann–Whitney U tests with Holm–Sidák correction for multiple comparisons using GraphPad Prism software.

### High-content cell morphology analysis

U87 cells were seeded into a 384-Well Nunc™ black plate with optically clear polymer bottom (Thermo Fisher, USA) at 1000 cells/well and with six replicates of each cell line. After 24 h, cells were gently washed three times with 1× PBS and stained with PhenoVue Cell Painting JUMP kit according to the manufacturer’s instructions (Revvity, USA) and then stored at 4°C in 50 µl of PBS until analysis by the Operetta CLS Imaging System (PerkinElmer, USA). Analysis was done with Harmony High-Content Imaging and Analysis Software ver4.8 (Perkin Elmer, USA).

### Intracranial GBM orthotopic xenograft

All animal use and care were in accordance with institutional guidelines and approved by the Animal Ethics Committee of South Australian Local Health Network (Approval 980–12). NOD.Cg-*Prkdc^scid^ Il2rg^tm1Wjl^*/SzJ (NSG) animals were purchased from Animal Resource Centre, and 10^5^ U87 cells expressing pCAG::luc-eGFP were stereotactically injected into 8-week-old NSG mice in 2 µl of HBSS with stereotactic instrument with robotic microinjection and drill (Neurostar). Bioluminescence imaging was performed at 3 and 5 weeks post-engraftment using IVISbrite D-Luciferin Potassium Salt (XenoLight, Revvity) at 150 mg/kg, intraperitoneally and imaged with IVIS Spectrum (PerkinElmer). Animals were humanely euthanized upon reaching predefined humane endpoints.

## Results

### CircRNAs are regulated in a cell type-dependent manner during human stem cell differentiation

H9 hESCs were differentiated into five cell populations—definitive endoderm (DE) [40], cardiomyocyte spheres (Cardio) [41], primitive hepatocytes (Liver) [42], floor plate precursors (FP) [40], and dopaminergic neurons (Dopa) [43]—as per published methods (Fig. 1B). Poly-A sequencing (mRNAseq) demonstrated that each differentiation protocol yielded cells expressing expected lineage markers (Fig. 1C).

**Figure 1:**
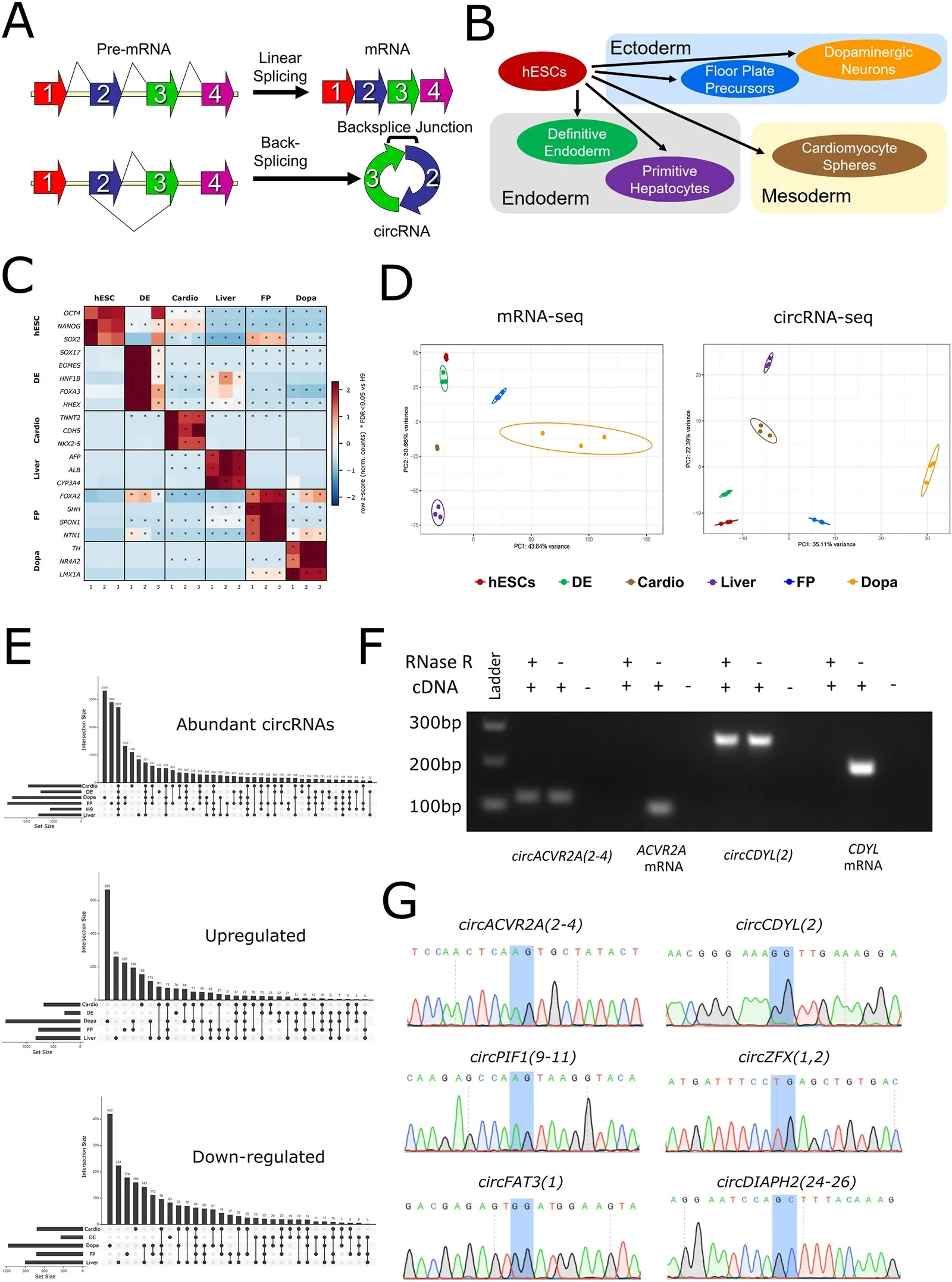
CircRNAs are abundant and regulated throughout hESC differentiation. (**A**) Linear splicing of pre-mRNA generates mRNA transcripts with sequential exons joined together, while backsplicing generates circRNAs whereby the downstream 3′ end of an exon is joined to the 5′ end of an upstream exon. (**B**) Schematic of differentiated cells that comprise the three germ layers ectoderm, mesoderm, and endoderm. (**C**) Abundance of canonical lineage-marker expression (RPKM, row *z*-scored) within each cell population from mRNA sequencing in three independent biological replicates (numbered along the bottom). Heatmap of canonical lineage-marker mRNA expression (RPKM, row *z*-scored) across H9 hESCs and five differentiated lineages, three biological replicates each. Colour, row *z*-score of normalized expression; * FDR < 0.05 versus H9 (DESeq2). (**D**) Principal component analysis (PCA) of individual samples following messenger RNA sequencing (mRNA-seq) and circRNA-seq showing grouping of replicates and stratification of distinct populations. (**E**) Upset plots presenting the total number of abundant circRNAs across three biological replicates of each differentiated cell population (top panel). The number of circRNAs shown to be upregulated (middle panel) and downregulated (lower panel) by differential expression analysis comparing cell populations with H9 hESCs. (**F**) Agarose gel electrophoresis of reverse transcriptase–polymerase chain reaction (RT-PCR) products for circRNAs and mRNAs following Ribonuclease R (RNase R) digestion. (**G**) Sanger sequencing chromatograms for RNase R-resistant RT-PCR amplicons. The two bases that comprise the BSJ are highlighted in blue.

To detect and enrich for circRNAs, RNA from each cell type underwent RNase R digestion and rRNA depletion followed by total RNAseq, with circRNAs identified using CIRCexplorer2 [28]. While over 100 000 unique circRNAs were identified, high-stringency thresholds of two unique BSJ reads per circRNA and presence in all replicates, found 2215–6175 circRNAs in each cell type (Supplementary Table S1 and Supplementary Fig. S1A). PCA of these data demonstrates clear stratification of cell types using either circRNA or mRNA datasets (Fig. 1D). Agreeing with previous reports for brain-derived circRNAs, the neural lineages (FP and Dopa) showed the greatest abundance of unique circRNAs (Fig. 1E) [44].

Applying differential expression analysis (edgeR), 91 circRNAs were commonly and significantly differentially expressed (*P* < .05) from undifferentiated hESCs to all five cell lineages, with 27 circRNAs increased and 64 circRNAs decreased (Fig. 1E, Supplementary Table S1, and Supplementary Fig. S1B). However, the majority of differentially expressed circRNAs (3.5%–14.2%) are regulated in a more cell-type-specific manner. While positive correlations between circRNA and cognate mRNA abundance were evident for the top 100 abundant circRNAs (coefficient of determination 0.41–0.62), numerous circRNAs showed cell-type-specific dysregulation from their cognate mRNA (Supplementary Fig. S1C and D).

### Identification of candidate circRNAs for functional validation

CircRNA candidates were chosen for targeted knockdown according to the following key criteria—commonly upregulated (i.e. *circCDYL*(2), *circZFX*(1,2)*, circACVR2A*(2–4)), commonly downregulated (i.e. *circPIF1*(9-11), *circDIAPH2*(24-26)*, circDNMT3B*(7-9)), and cell-type-specific (i.e. *circMYBPC3*(6-9), *circSUCO*(2-9), *circFAT3*(1))—resulting in a total of 31 circRNAs. RT-PCR on RNase R-treated RNA, followed by Sanger sequencing, confirmed each candidate as a *bona fide* circRNA (Fig. 1F and G).

### Knockdown of circRNAs by shRNAs induces stem cell differentiation

Two shRNAs were designed to target the candidate circRNA BSJ (Fig. 2A) and cloned into the *pLKO.1 puro* lentiviral plasmid. Two control lines were generated in H9 hESCs—empty vector (pLKO.1 puro) and a published NT shRNA sequence [45]—which both lacked visible phenotypic effects (Fig. 2B). Phenotypic analysis of the stable circRNA knockdown lines showed morphological effects for six circRNAs (Fig. 2B). Interestingly, of the 25 silenced circRNAs yielding no phenotype, 24 were lowly expressed in H9 cells (<3 copies/cell), while the six circRNAs eliciting a phenotype were present at 19–355 copies/cell (Fig. 2C and Supplementary Fig. S2A). RT-qPCR relative to *YWHAZ*, validated as the optimal reference transcript across hESC differentiations, demonstrated target circRNA knockdown efficiencies ranging from 33% to 99% (Fig. 2D and Supplementary Fig. S2B and C).

**Figure 2.**
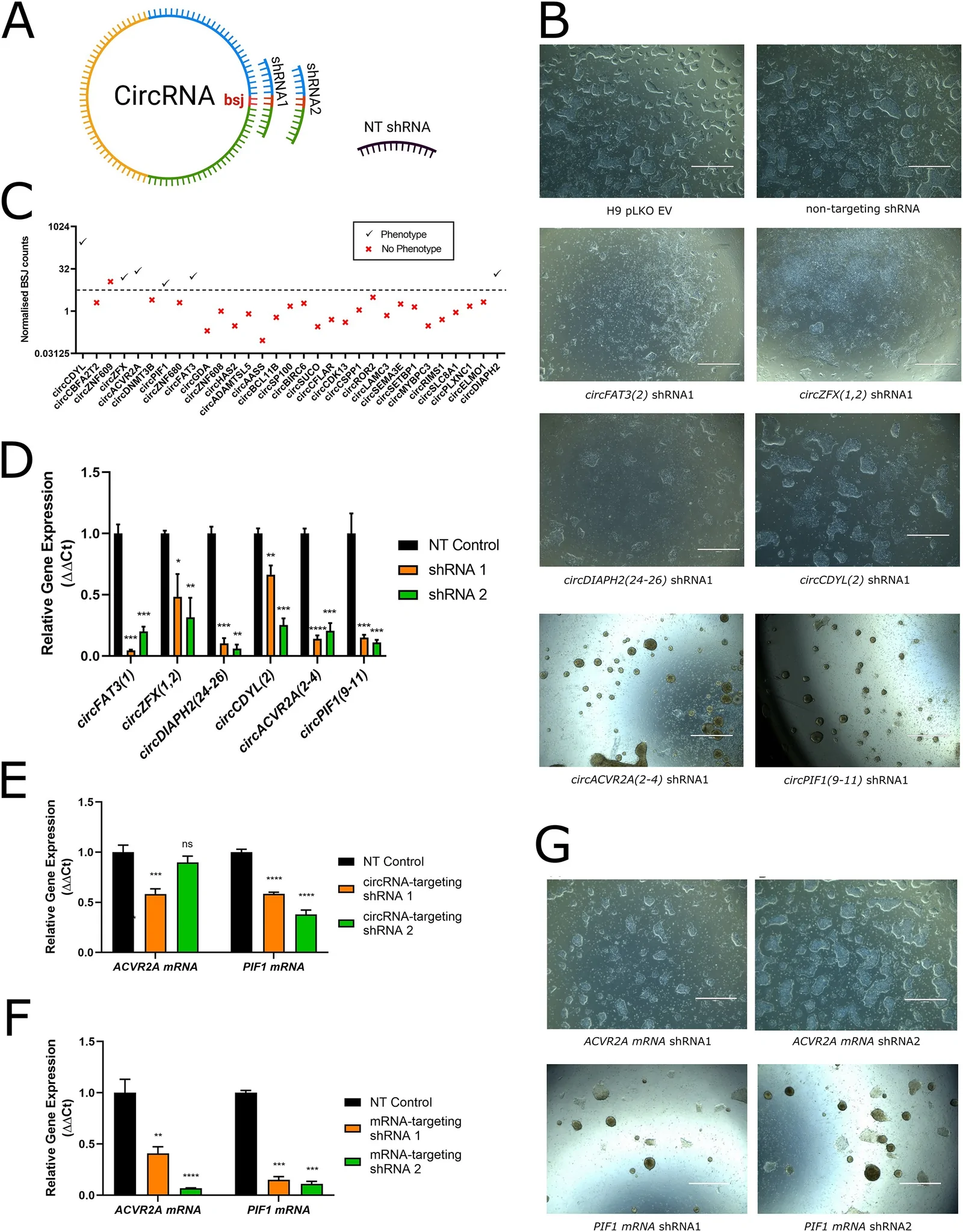
shRNA knockdown of candidate circRNAs induces phenotypic alterations in H9 hESCs. (**A**) Illustration of design for two shRNAs to target the BSJ of candidate circRNAs in hESCs, with a NT shRNA control. Created in BioRender. Conn, S. (2026) https://BioRender.com/ilp9n7j. (**B**) Brightfield microscopy images of hESC culture stably transduced with one shRNA targeting a specific circRNA after 7 days post-selection in puromycin. 4× magnification, scale bar = 1 000 µm. (**C**) Normalized expression levels for each circRNA (BSJ counts per 10 million mapped reads) indicating whether a phenotype was observable (tick) or no phenotype (red cross) following shRNA knockdown. Dotted line indicates an expression level of three circRNA copies per cell as determined by absolute quantitative RT-PCR. (**D**) Relative expression of six circRNAs in hESC lines stably expressing either NT control shRNA or each of two circRNA-specific shRNAs (shRNA1, shRNA2) as quantified by RT-qPCR. (*n* = 3 biological replicates; Mean + SD). (**E**) Relative expression of *ACVR2A* and *PIF1* mRNA in hESC lines stably expressing either NT control shRNA or each of two shRNAs specific for *circACVR2A*(2–4) and *circPIF1*(9-11), respectively (shRNA1, shRNA2), as quantified by RT-qPCR (*n* = 3 biological replicates; Mean + SD). (**F**) Relative expression of *ACVR2A* and *PIF1* mRNA in hESC lines stably expressing either NT control shRNA or each of two shRNAs specific for *ACVR2A* and *PIF1* mRNA, respectively (shRNA1, shRNA2), as quantified by RT-qPCR. (*n* = 3 biological replicates; Mean + SD). (**G**) Brightfield microscopy images of lentiviral-transduced hESC culture stably expressing each of two shRNAs targeting *ACVR2A* (top panels) and *PIF1* (bottom panels) mRNA, 7 days post-selection in puromycin. 4× magnification, scale bar = 1 000 µm. Statistical analysis was performed using unpaired *t*-tests, not correcting for multiple comparisons. (****P < .0001, ***P < .001, **P < .01, *P < .05, ns: not significant).

Critically, phenotypic effects were consistent for both shRNAs for each circRNA (Fig. 2B and Supplementary Fig. S2D). These phenotypic effects grouped into two major categories with *circFAT3*(1)*, circCDYL*(2), *circZFX*(1–2), and *circDIAPH2*(24-26) knockdown showing loss of intact stem cell colonies and defined edges (Fig. 2B). In contrast, knockdown of *circACVR2A*(2–4) and *circPIF1*(9-11) induced spontaneous detached aggregate formation in H9 hESCs (Fig. 2B and Supplementary Fig. S2D) and H1 hESCs (Supplementary Fig. S2E). One of the two shRNAs used to silence *circACVR2A*(2–4) reduced the abundance of *ACVR2A* mRNA (NCBI Gene ID 92) (Fig. 2E), yet neither shRNA impacted protein abundance (Supplementary Fig. S2F). Furthermore, shRNA-mediated silencing of *ACVR2A* mRNA did not induce this aggregate phenotype, while *PIF1* mRNA silencing phenocopied the *circPIF1*(9-11) silencing (Fig. 2F and G). Critically, this phenotype was rescued by overexpression of *circACVR2A*(2–4) using the CircRNA mini vector, which introduces a molecular scar at the BSJ, precluding silencing by the shRNAs (Supplementary Fig. S2G) [46]. No visible differentiation was seen in H9 hESCs with 12.5-fold overexpression of *circACVR2A*(2–4) with NT or *circACVR2A*(2–4) shRNA silencing, while the empty vector line exhibited detached aggregate formation upon *circACVR2A*(2–4) knockdown (Supplementary Fig. S2H and I). Furthermore, the abundance of two representative pluripotency (*OCT4, NANOG*) and neuroectoderm markers (NES, *MAP2*) was not statistically different between the rescued line with circACVR2A(2–4) shRNA and the empty vector line expressing the NT shRNA (Supplementary Fig. S2J). Taken together, these results demonstrated that silencing of *circACVR2A*(2–4) (CIRCpedia v3: CIRCHSA_ACVR2A_31; circBase: *hsa_circ_0 001 073*; chr2:147896300–147899898) was responsible for cell differentiation and was prioritized in future experiments.

### Neuroectodermal differentiation of hESCs from *circACVR2A*(2–4) knockdown

*CircACVR2A*(2–4) is the most abundant circRNA from the *ACVR2A* locus (Fig. 3A and Supplementary Table S1). RNA fluorescence *in situ* hybridization (RNA-FISH) with a probe targeting the BSJ of *circACVR2A*(2–4) identified multiple foci localized to both the nucleus and cytosol of H9 hESCs (Fig. 3B).

**Figure 3.**
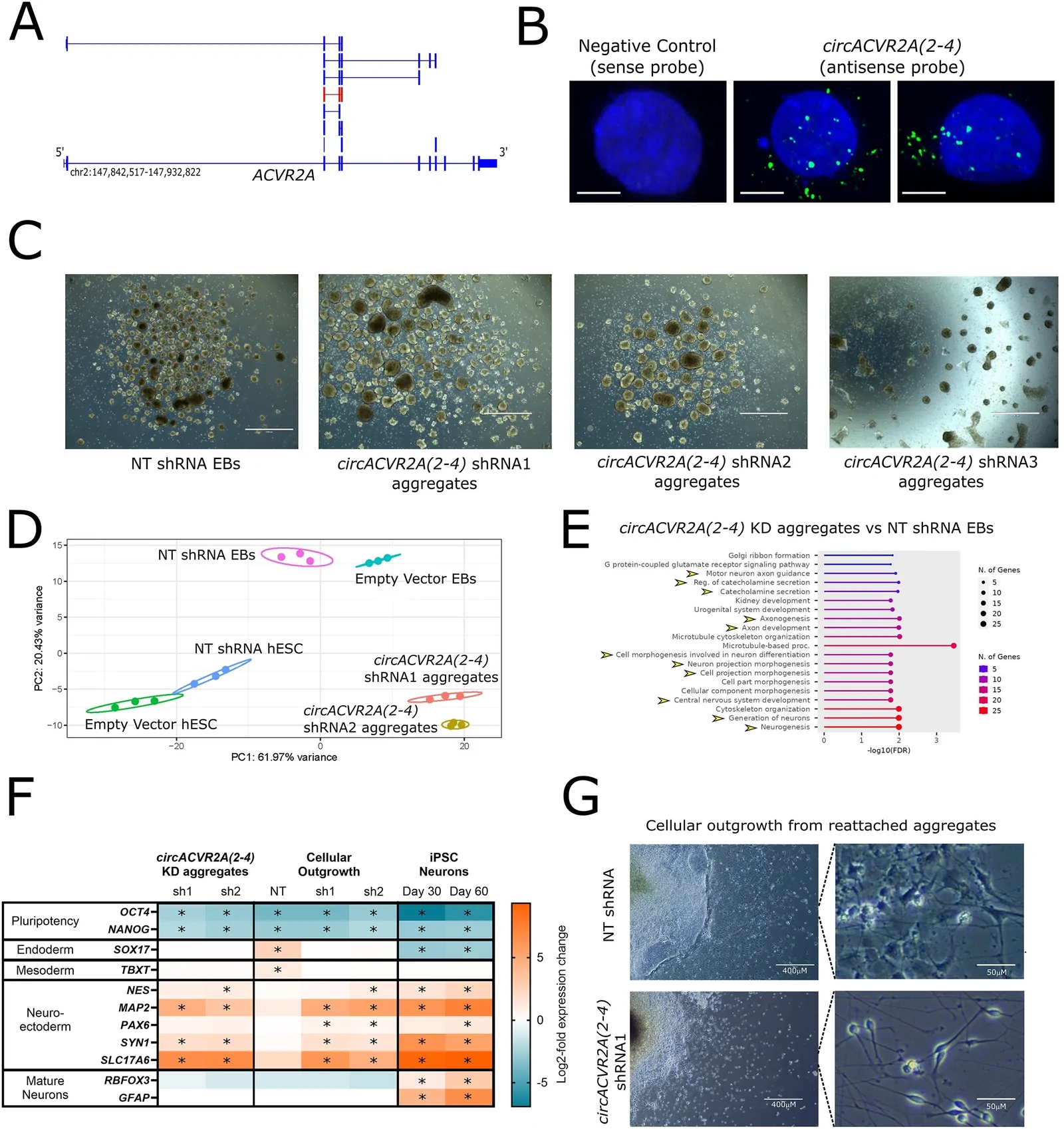
Silencing of *circACVR2A*(2–4) induces neuroectoderm differentiation of hESCs. (**A**) Schematic of identified circRNAs from the human ACVR2A gene from *circBase* and *CIRCpedia v2. ACVR2A* gene locus shown at bottom, with vertical blue lines representing exons. Blue lines represent individual circRNAs, with *circACVR2A*(2–4) highlighted in red. (**B**) Three-dimensional RNA-FISH in H9 hESCs showing localization of *circACVR2A*(2–4) in cytosol and nucleus. Negative control (sense probe, *left panel*) and *circACVR2A*(2–4) antisense probe (right panels) labelled with AlexaFluor 488 (green) and DAPI nuclear stain (blue). Magnification 40×. Scale bars, 5 µm. (**C**) Images of aggregates generated spontaneously from *circACVR2A*(2–4) silencing by shRNAs after 7 days or by culturing NT shRNA line under non-adherent conditions in StemFlex media for 72 h to produce size-matched embryoid bodies (EBs). 4× magnification, scale bar = 1000 µm. (**D**) PCA plot for *n* = 3 biological replicate mRNA-seq libraries harvested at 7 days post-transduction. (**E**) Gene Ontology analysis of differentially expressed RNA transcripts in *circACVR2A*(2–4) knockdown compared with NT shRNA (control) aggregates after 7 days post-transduction. Arrows highlighting neuroectoderm-linked ontologies. (**F**) Heatmap of RT-qPCR of pluripotency and lineage markers compared to NT EBs for hESC aggregates and cellular outgrowth from two shRNAs silencing of *circACVR2A*(2–4) (14 days post-transduction) aggregates and cells outgrown from NT EBs after reattachment to GelTrex as seen in panel (G). Students *t*-tests were employed to test for significant difference from hESCs, with correction for multiple comparisons using the Benjamini–Hochberg method; * FDR < 0.05, *n* = 3 replicates. (**G**) *CircACVR2A*(2–4) shRNA1 silencing-induced aggregates attached on Geltrex-coated dishes produce neural-like cells compared to NT shRNA EBs that display various cellular phenotypes. Right panel shows 8× magnification of left panel image focusing on outgrown cells.

The *circACVR2A*(2–4) knockdown lines phenocopied EBs generated from single-cell plating of asynchronous hESCs in low-attachment conditions [47] (Figs 2B and 3C). To explore transcriptional signatures of differentiation, mRNA-seq was performed on control EBs (3-day timepoint) and size-matched *circACVR2A*(2–4) knockdown-induced aggregates (7-day timepoint) grown in low-attachment conditions. In accordance with the noted phenotypic differences, both *circACVR2A*(2–4) shRNA lines clustered together in PCA (Fig. 3D) and were stratified from NT shRNA and empty vector (pLKO.1) EBs (which also stratified from their adherent hESC culture equivalents). This suggested that, even within these early embryonic aggregates, *circACVR2A*(2–4) silencing induces distinct cellular differentiation gene expression signatures (Supplementary Fig. S3A). Gene Ontology analysis of the top 100 most upregulated RNA transcripts in *circACVR2A*(2–4) shRNA treatments compared with NT shRNA EBs reveals an enrichment for several NE biological processes (Fig. 3E). This was also apparent when comparing to hESC adherent colonies, where 12 of the top 20 enriched ontologies were NE process-related, while NT shRNA EBs of similar size and at the same timepoint lacked these enriched ontologies (Supplementary Fig. S3B).

Consistent with differentiation, a reduction in *POU5F1*/*OCT4* and *NANOG* stem cell markers were observed in *circACVR2A*(2–4) aggregates compared with NT shRNA hESCs (Fig. 3F and Supplementary Fig. S3C). Increase in endoderm (*SOX17*) and mesoderm (*TBXT*) markers were only found for NT shRNA cells outgrown from EBs made from this line (Fig. 3F and G). NE marker *NES* was significantly increased in the differentiating aggregates and cells outgrown from *circACVR2A*(2–4) shRNA-silenced lines. Increases in other NE markers, including synaptic markers SYN1 and SLC17A6, somatodendritic marker MAP2, and PAX6, suggest that synaptic processes may be increased, and glutamatergic neurons may be one of several neuronal cell types within the *circACVR2A*(2–4)-silenced aggregates (Fig. 3H). However, no change in *RBFOX3*/*NeuN* and an absence of GFAP expression indicate that these aggregates lack mature neurons and astrocytes compared to day 30 and day 60 iPSC-derived neurons.

When NT shRNA EBs were allowed to attach on GelTrex-coated plates, multiple cell types, including fibroblast- and neural-like cells, were observed emanating from control EBs (Fig. 3G). Contrastingly, the cells emanating from *circACVR2A*(2–4) knockdown aggregates almost exclusively displayed a neuronal morphology with clear dendrite projections and central cell bodies. RT-qPCR showed reduced pluripotent gene expression and stimulation all three lineage markers in control EBs, but loss of endoderm and mesoderm markers and gain of NE markers in circACVR2A(2–4)-silenced cells (Fig. 3F). Taken together, *circACVR2A*(2–4) knockdown enriches for a population of NE-derived cells both transcriptionally and phenotypically.

### *CircACVR2A*(2–4) silencing does not perturb the expression networks of predicted miRNA interactors

Dong *et al*. [48] found *circACVR2A*(2–4) bound four human miRNAs, hsa-mir-626, hsa-mir-1265, hsa-mir-1279, and hsa-mir-1200, in bladder cancer cell lines, derepressing target genes including *EYA4* (Supplementary Fig. S4A). To explore this mechanism in *circACVR2A*(2–4)-silenced hESCs, we exploited miRDB [49] to select the top five highest-ranked mRNA targets for each of these four miRNAs. The mRNA-seq of *circACVR2A*(2–4) knockdown hESCs did not reveal significant or consistent differences in mRNA expression for any of these four miRNAs compared to the NT control shRNA (Supplementary Fig. S4B). This indicates that miRNA sponging is likely not a significant contributor to the phenotype of *circACVR2A*(2–4) knockdown in hESCs. While three open reading frames were predicted for *circACVR2A*(2–4) using circPrimer2.0, no evidence was found for translation or ribosomal engagement from the riboCIRC database comprising over 3000 published ribo-seq datasets [50].

### *CircACVR2A*(2–4) interacts with cyclin H without affecting cell cycle progression

To assess functional mechanisms by which *circACVR2A*(2–4) silencing drives NE differentiation, native circRNA pulldown followed by protein mass spectrometry was performed (Fig. 4A) [33]. To increase circRNA and protein capture, *circACVR2A*(2–4) was overexpressed in HEK293 cells (60-fold, Fig. 4B). Interactors were identified by the presence of the protein in all three replicates and absence from any of the three replicates of the sense control pulldown (Supplementary Table S2). Under these stringent criteria, five proteins were identified as interactors of *circACVR2A*(2–4) (Fig. 4C and Supplementary Fig. S4C). Interestingly, cyclin H (CCNH), a member of the CAK complex, known to be involved in cell cycle progression and transcriptional regulation by phosphorylating RNAPII, was found to have the highest coverage, despite its small molecular mass. Interestingly, both other components of the CAK complex, CDK7 and MAT1, were found in two of three antisense replicates.

**Figure 4.**
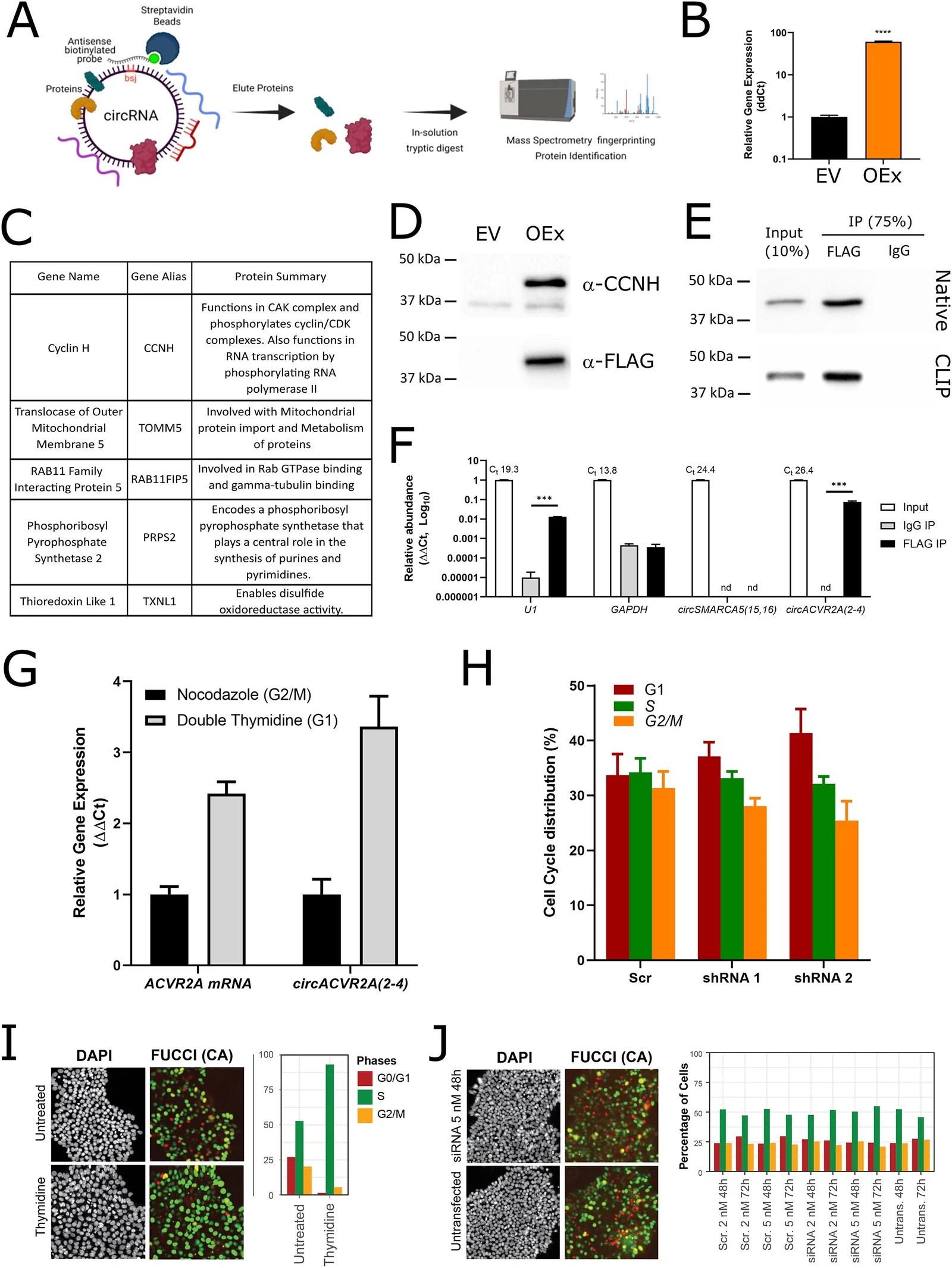
*CircACVR2A*(2–4) interacts with cyclin H without affecting cell cycle progression. (**A**) CircRNA-specific pulldown and mass spectrometry to identify *circACVR2A*(2–4)-bound proteins. Created in BioRender. Conn, S. (2026) https://BioRender.com/an0gywn. (**B**) Overexpression of *circACVR2A*(2–4) in HEK293T cells. Statistical analysis was performed with an unpaired one-tailed *t*-test. (**** = *P* < .0001) (*n* = 3) (Mean + SD). (**C**) Proteins identified from *circACVR2A*(2–4) pulldown and mass spectrometry, which were detected in all three *circACVR2A*(2–4) sense pulldowns and absent from all antisense pulldowns. (**D**) Western blot detection of CCNH-FLAG overexpression in HEK293T cells, with anti-CCNH and Anti-FLAG antibodies. (**E**) Native IP (top) and UV-crosslinked IP (CLIP, bottom) of CCNH-FLAG with anti-FLAG (mouse) antibody after overexpression of *circACVR2A*(2–4) and CCNH-FLAG. Western blot detection with anti-FLAG (rabbit) antisera. (**F**) Enrichment of *circACVR2A*(2–4) and control RNA U1, but not *GAPDH* mRNA or *circSMARCA5* [15, 16], with FLAG CLIP of CCNH in HEK293T cells. RT-qPCR presented relative to input; mean + SD (*n* = 3 replicates), Students *t*-test (*** *P* < .001), nd: not detected. (**G**) RT-qPCR demonstrating expression of *circACVR2A*(2–4) and *ACVR2A* mRNA in hESC populations following nocodazole treatment (G2/M block, *black filled*) and double thymidine treatment (G1/S block, grey filled). (**H**) Cell cycle analysis of NT shRNA EBs and *circACVR2A*(2–4) knockdown-induced aggregates. Statistical analysis was performed with a two-way analysis of variance (ANOVA) with Dunnett’s test for correcting for multiple comparisons (all comparisons with Scr control were not-significant) (*n* = 6) (Mean + SD). FUCCI cell cycle reporter system in H9 hESCs showing (**I**) successful G1/S arrest with double thymidine block (thymidine). Fluorescence microscope images showing DAPI stain and confocal imaging in green, yellow, and red channels. Graph shows data from >500 cells per condition. (**J**) Cell cycle distribution (percentage of cells) of H9-FUCCI cells untransfected, or transfected with scrambled siRNA control (Scr) or siRNA #2 for *circACVR2A*(2–4) at 2 and 5 nM after 48 or 72 h.

To validate this association, a FLAG-tagged CCNH construct was co-expressed in HEK293T cells (Fig. 4D) with *circACVR2A*(2–4). Successful enrichment of CCNH-FLAG was seen following UV-crosslinked immunoprecipitation and native immunoprecipitation with FLAG antisera (Fig. 4E). A positive control RNA for CCNH CLIP is U1 small nuclear RNA [51], which was found to be enriched in the FLAG IP. Importantly, very low levels of negative control mRNA (*GAPDH*) and undetectable levels of negative control circRNA, *circSMARCA5*(15,16) (CIRCpedia v3: CIRCHSA_SMARCA5_5; hsa_circ_0 001 445; chr4:143543508–143543972), sharing nuclear and cytoplasmic distribution with *circACVR2A*(2–4) but ∼4-fold higher abundance, were seen in either IgG or FLAG IPs (>1000-fold less than *circACVR2A*(2–4)). Importantly, in overexpression conditions, *circACVR2A*(2–4) was uniquely detectable in the FLAG CLIP (7.4% of input) and not in the IgG pulldown, confirming the interaction between CCNH and *circACVR2A*(2–4) (Fig. 4F).

Spatiotemporal expression profiling for *circACVR2A*(2–4) was performed to ensure the subcellular localization and timing of expression coincided with the putative targets. Nuclear/cytosolic fractionation demonstrated 32% and 15% of *circACVR2A*(2–4) are localized to the nucleus of H9 hESCs and HEK293T cells, respectively (Supplementary Fig. S4D). This is in agreement with the RNA-FISH results (Fig. 2B) and is similar to published data in bladder cancer cell lines, T24 and UM-UC-3 [48]. Based on the per-cell abundance calculated by absolute RT-qPCR (Supplementary Fig. S2A), this would equate to ∼52 *circACVR2A*(2–4) transcripts per hESC nucleus.

Analysing the expression of *circACVR2A*(2–4) across the cell cycle was achieved via hESCs synchronization using (i) double thymidine treatment (G1/S, S block) and (ii) 0.2 µg.ml^−1^ nocodazole treatment for 24 h (G2/M block). C*ircACVR2A*(2–4), along with its cognate mRNA, was most highly expressed in the population of cells enriched in G1 (Fig. 4G), in accordance with the role of CCNH. A more detailed time-course of expression was carried out in a non-pluripotent cell line, human A549 lung adenocarcinoma cells, where 48-h serum starvation and release were applied to study the expression of *circACVR2A*(2–4) throughout the cell cycle at 2-hourly intervals. These results support the significant enrichment of *circACVR2A*(2–4) in the G1 phase within these cells (Supplementary Fig. S4E–G).

Inhibition of the CAK complex has been shown to be required for progression of cells through G1 phase of the cell cycle [52], and significant duration and checkpoint remodelling of G1 phase are functionally important for differentiation, particularly late G1 phase lengthening in promoting NE differentiation [53, 54]. To identify cell cycle-related consequences, NT shRNA hESCs and the two shRNA knockdown *circACVR2A*(2–4) aggregate lines underwent cell cycle analysis by flow cytometry. In both *circACVR2A*(2–4) knockdown lines, no significant changes were seen across phases of the cell cycle (Fig. 4H).

As a third strategy to assess cell cycle perturbation with *circACVR2A*(2–4) knockdown, siRNAs targeting this circRNA were transfected into H9 hESCs stably expressing the Fluorescence Ubiquitin Cell Cycle Indicator (FUCCI(CA)) reporter construct. This FUCCI(CA) construct dynamically and fluorescently marks cells in G1 (red), G1/S and S (green), and G2/M phases (orange) [55]. Double thymidine block validated the FUCCI(CA) system by arresting cells in S and G1/S phases (Fig. 4I). Utilizing *circACVR2A*(2–4) siRNA #2, which achieved the greatest knockdown (>80%), resulted in no significant change in cell cycle distribution at two different doses (2 and 5 nM) and timepoints (48 and 72 h) (Fig. 4J).

### *CircACVR2A*(2–4) silencing causes a decrease in CAK phosphorylation of RNA polymerase II, but not CDKs

Sandwich ELISA assays were employed to quantify total CDK1, CDK2, CDK4, and CDK7 and phosphorylated CDK1^Thr161^, phosphorylated CDK2^Thr160^ (both within the T-loop region and catalysed by the CAK complex [56]), and phosphorylated CDK1^Thr14^ (not catalysed by CAK, but by PKMYT1 [57]). Concordant with the lack of cell cycle distribution defects, the abundance of each CDK and the relative proportion of phosphorylated CDK1 or CDK2 in *circACVR2A*(2–4) knockdown lines were not significantly altered compared to EBs made from NT shRNAs (Fig. 5A). In addition, no quantitative effect on phospho-substrates of CDK proteins was found (Fig. 5B).

**Figure 5.**
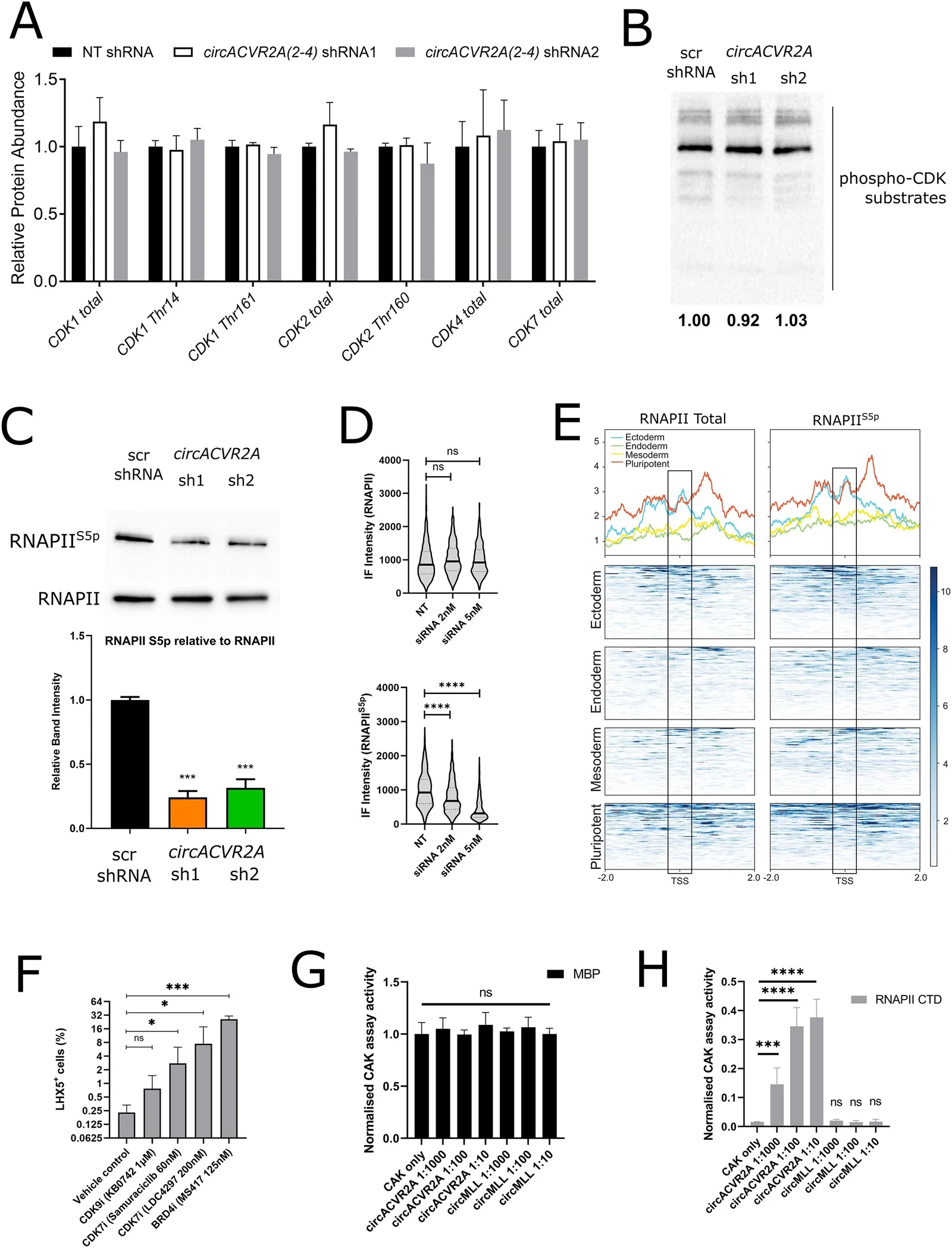
*CircACVR2A*(2–4) affects the phosphorylation of RNAPII by the CAK complex. (**A**) Sandwich immunoassay (AlphaLISA SureFire Ultra) showed no significant difference between CDKs and phospho-CDKs in hESCs comparing NT shRNA EBs and *circACVR2A*(2–4) shRNA1 and shRNA2 aggregates. (**B**) Western blot with phospho-CDK substrate antisera on hESCs without (scrambled shRNA control) and with KD of *circACVR2A*(2–4) (two shRNAs). Quantification using total protein loading written below blot. (**C**) Western blot for total RNAPII and RNAPII phosphorylated on serine 5 of CTD (RNAPII S5p). Quantification of RNAPII S5p, normalized by total protein loading and presented relative to total RNAPII (lower panel). *N* = 3 replicates, Mean + SD, Student’s *t*-test (*** *P* < .001). (**D**) Quantitative immunofluorescence for RNAPII and RNAPII^S5p^ in H9 hESCs transfected with NT siRNA and *circACVR2A*(2–4) siRNA #2 at 2 and 5 nM. *n* = 500 cells per condition, with violin plot showing IF intensity per cell. (**E**) ChIP-Seq intensity map (top panel) and heatmap for occupancy of top 100 lineage genes for pluripotent (red trace), ectoderm (blue), endoderm (green), and mesoderm (yellow) lineages in a 4 kb window surround the TSS. (**F**) Impact of specific inhibitors for CDK9 (KB0472), CDK7 (samuraciclib, LDC4297), and BRD4 (MS417) inhibitors on driving NE differentiation as defined by LHX5+ cells (%) on H9 hESCs after 48 h with vehicle control [DMSO, 0.1% (v/v)]. Mean + SEM, *n* = 3 biological replicates. One-way ANOVA (ns: not significant, * *P* < .05, ***P < .001). *In vitro* CAK activity assay using recombinant CAK protein and (**G**) MBP or (**H**) RNAPII CTD peptide. Assay activity quantified by luminescence and normalized to protein input, relative to CAK-only control activity on MBP. *In vitro* synthesized and purified *circACVR2A*(2–4) and *circMLL*(9,10) were co-incubated with CAK protein (30 ng) at molar ratios, 1:1000, 1:100, and 1:10. *N* = 6 replicates, Mean ± SD. Paired *t*-tests performed (**** *P* < .0001; *** *P* < .001, ns: not significant).

Uniquely, the metazoan CAK complex has a critical secondary function beyond CDK activation, in that it associates with basal transcription-repair factor TFIIH, which directly phosphorylates RNAPII on its C-terminal repeat domain at serine position 5 (RNAP^S5p^) [58, 59]. This modification is widely considered to control the transition from transcription initiation to pausing, with RNAPII remaining in a poised state [60, 61]. Therefore, we quantified the abundance of total RNAPII and RNAPII^S5p^ by western blotting. Despite no change in total RNAPII levels, RNAPII^S5p^ decreased 76% and 68% in shRNA1 and shRNA2 knockdown lines (*P* = .000144 and *P* = .000658, Student’s *t*-test), respectively, compared to NT shRNA control hESCs (Fig. 5C). This was also assessed on a per-cell basis by performing quantitative immunofluorescence, where a significant decrease in RNAPII^S5p^ signal was found with *circACVR2A*(2–4) knockdown at 2 and 5 nM siRNA doses, with no significant effect on total RNAPII levels (Fig. 5D and Supplementary Fig. S5A and B).

As phosphorylation of Ser5 residues in the CTD of RNAPII is a mark associated with paused transcription [62], investigation of RNAPII^pSer5^ occupancy on the hESCs was completed. Probing published RNAPII chromatin immunoprecipitation sequencing (ChIP-Seq) data in hESCs, we focused on the top 100 genes specific to pluripotent, ectoderm, endoderm, and mesoderm cells based on single cell RNA-Seq data from differentiated hESCs [37] (Supplementary Table S3). RNAPII and RNAPII^S5p^ occupancy immediately flanking the TSS was highest for ectoderm genes, followed closely by pluripotent genes, with a local peak ~100 nt into the transcript (Fig. 5E). Both gene subsets demonstrate a two-fold higher occupancy at this local maximum compared with endoderm and mesoderm gene sets (Fig. 5E). This indicates that pluripotent and ectoderm genes are poised for transcription release upon removal of the S5p mark, which is associated with *circACVR2A*(2–4) silencing. In support of this, strong correlation was found between the transcripts upregulated following *circACVR2A*(2–4) silencing, whereby 74% of RNAPII^S5p^-occupied ectoderm genes were upregulated at the mRNA level compared to hESCs, 52% for pluripotent gene transcripts, but only 19% and 23% of the endoderm and mesoderm gene sets were upregulated, respectively.

To further investigate whether the CAK complex is linked with NE differentiation, H9 hESCs were exposed to specific inhibitors of CDK7 (samuraciclib, LDC4297) as regulators of CAK activity and RNAPII^S5p^, CDK9 (KB0742) as an inhibitor of RNAPII^S2p^ and BRD4 (MS417) as epigenetic reader of acetylated histones, with the latter known to induce NE differentiation in stem cells [63]. MS417 was found to induce an average of 26% of H9 hESCs to express the NE marker LHX5, by immunofluorescence (*P* < .001; Fig. 5F and Supplementary Fig. S5C). Both CDK7 inhibitors showed a significant increase in LHX5^+^ H9 hESCs (*P* < .05), while higher dose KB-0742 (1 µM) did not significantly increase NE differentiation (Fig. 5F). This provides further evidence of the importance of CDK7 within the CAK complex in regulating RNAPII^S5p^ and NE differentiation.

While reduced cellular RNAPII^S5p^ correlated with the knockdown of *circACVR2A*(2–4), we sought to probe whether the interaction between *circACVR2A*(2–4) and CCNH was causative of altered RNAPII phosphorylation. To assess whether CAK activity against RNAPII was affected *circACVR2A*(2–4) was *in vitro* synthesized [20] and co-incubated with human recombinant CAK complex. Kinase activity of the ternary CAK complex was quantified across two distinct substrates—(i) MBP to assess general kinase activity and (ii) a peptide based on the CTD of RNAPII (YSPTSPSYSPTSPSYSPTSPSKKKK) to differentiate the activities of the CAK complex. At baseline, the activity of the CAK complex against MBP was 100-fold greater than that against the RNAPII CTD peptide (Fig. 5G and H). At three concentrations of *circACVR2A*(2–4) (achieving a molar ratio of 1:1000,1:100, and 1:10), there was no effect on MBP phosphorylation (Fig. 5G). However, co-incubation significantly increased activity against the RNAPII CTD peptide, even at the lowest dose (Fig. 5H). Importantly, addition of *circMLL*(9,10), which we have previously shown is unable to bind any protein member of the CAK complex or RNAPII [30], did not affect CAK activity (Fig. 5G and H). This implicates *circACVR2A*(2–4) in playing a key role in regulating RNAPII^S5p^.

### Neuronal cell migration is reduced with *in vivo* silencing of *circACVR2A*(2–4)

Of the three identified circRNAs from the mouse gene orthologue, *Acvr2a* (NCBI Gene ID 11480), from CIRCpedia, we identified the putative orthologue of *circACVR2A*(2–4), composed of exons 2–4 (CIRCpedia v3: CIRCMMU_Acvr2a_1; circBase: *mmu_circ_0 001 017*; chr2:48870288–48873473). Comprising the same length (473 nt) and possessing 95% sequence identity (Supplementary Fig. S6A), this maximizes the potential for overlapping functionality. Confirmed as a *bona fide* circRNA (Supplementary Fig. S6B), digital PCR demonstrated that *mmu_circAcvr2a*(2–4) is over eight-fold more abundant in brain tissue than other tissues (Fig. 6A). Furthermore, the abundance of *mmu_circAcvr2a*(2–4) was highest in the hippocampus of the adult brain, a primary source of neural stem cells within the dentate gyrus (Fig. 6B). Given the role of *circACVR2A*(2–4) in hESCs, we quantified its expression in embryonic and postnatal mouse brain tissue and found limited variability in expression of *mmu_circAcvr2a*(2–4) between embryonic day 13 until postnatal day 8 despite a gradual increase in *mmu_Acvr2a* (Fig. 6C).

**Figure 6.**
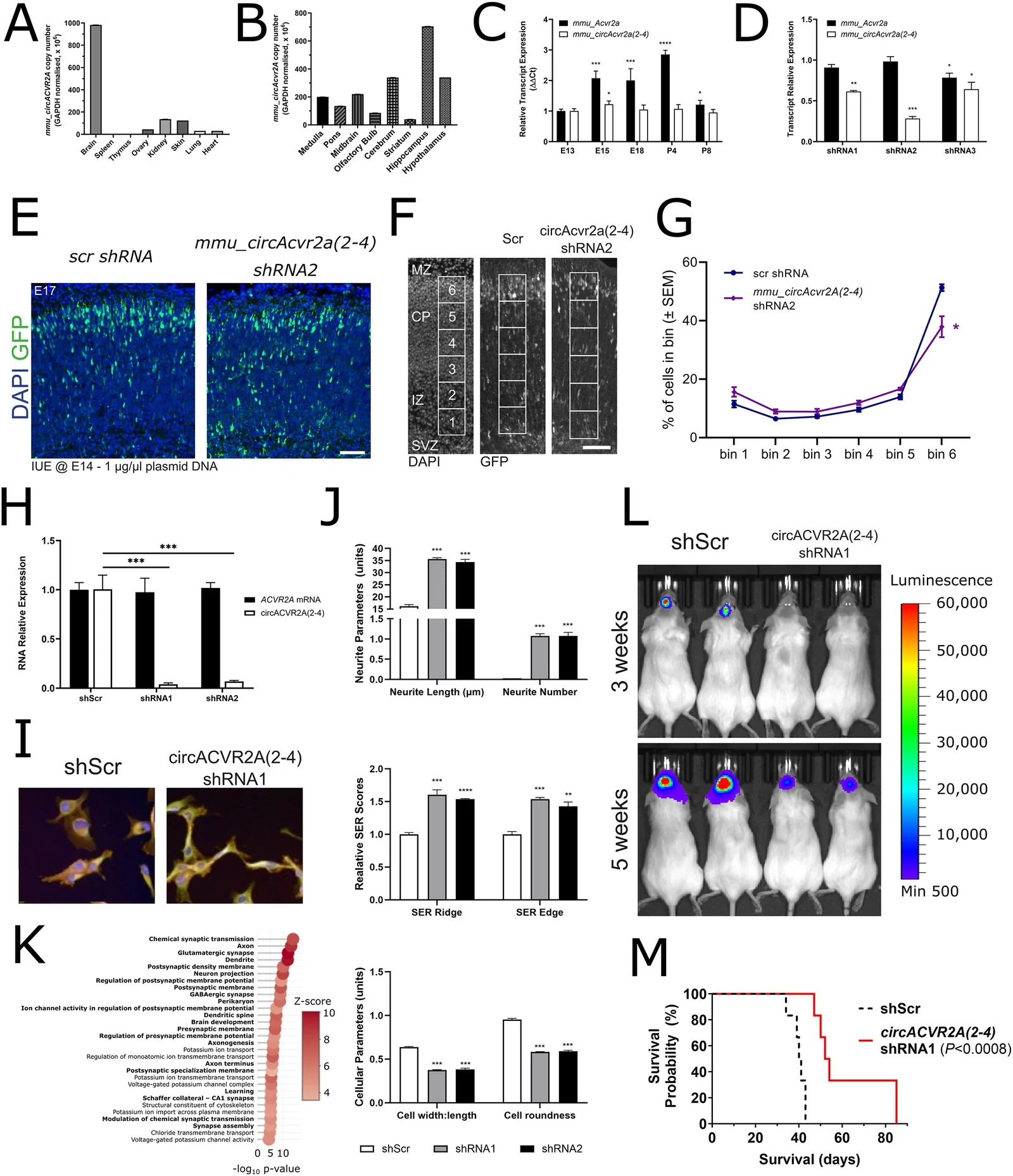
*CircACVR2A*(2–4) knockdown reduces cortical neuron migration and GBM tumour growth *in vivo*. (**A**) Sanger sequencing chromatogram of *mmu_circAcvr2a*(2–4) amplicon showing BSJ between exons 4 and 2. (**B**) Digital PCR quantifying copies of *mmu_circAcvr2a*(2–4) normalized to *GAPDH* in tissues of 8-week-old mice. Mean ± SD (*n* = 3 replicates). (**C**) RT-qPCR amplification of *mmu_circAcvr2a*(2–4) and *mmu_Acvr2a* mRNA from different developmental stages of C57Bl6 mice brain cortex. RT-qPCR data presented as relative expression of transcripts, mean + SD, normalized to *GAPDH. N* = 3 biological replicates. (**D**) RT-qPCR effect of three shRNAs designed against the BSJ of *mmu_circAcvr2a*(2–4) in NIH3T3 cells, 48 h post-transfection and GFP sorting of transfected cells by flow cytometry. (**E**) Embryonic day (E)17 brain cryosections of embryos *in utero* electroporated at E14 with 1 µg/µl scrambled (right panel) or short-hairpin vector *mmu_circAcvr2a*(2–4) (left panel). Vectors contained an IRES-GFP cassette. GFP staining was enhanced by immunohistochemistry, and sections were counterstained with DAPI. Scale bar = 50 μm. (**F**) To analyze neuron migration after electroporation, six-square bins were placed in each image to cover the intermediate zone (IZ, bin 1–2) and cortical plate (CP, bin 4–6). All GFP-positive migrating neurons in the bins were counted and percentage of cells per bin were averaged from three bins per image, from three sections per embryo. Scale bar = 50 μm. MZ = marginal zone, SVZ = subventricular zone. (**G**) Quantification of the percentage of cells per bin as in panel (F). Graphs represent mean ± SEM. * *P* < .05. In purple: *P* value SCR versus shRNA2. Mann–Whitney U test with Holm–Sidák correction for multiple comparisons (* *P* < .05). (**H**) RT-qPCR effect of two shRNAs designed against the BSJ of *circACVR2A*(2–4) in stable U87 cells. (**I**) High-content image of U87 cells with scrambled shRNA (shScr) and *circACVR2A*(2–4) shRNA 1 stained with PhenoVue™ Cell Painting JUMP kit and (**J**) analysis of phenotypic parameters (*n* = minimum 4500 cells) matching NE morphology with increased neurite length and number (top panel), increased SER Ridge and Edge scores (middle panel) and decreased cellular width:length and roundness (bottom panel). (**K**) Geneset enrichment analysis comparing U87 cells stably silenced for *circACVR2A*(2–4) compared to *shScr. N* = 3 biological replicates, with NE-related ontologies in bold. (**L**) Bioluminescence imaging of engrafted tumours from two representative animals for shScr and *circACVR2A*(2–4) shRNA 1 groups at 3 weeks (top panel) and 5 weeks (bottom panel) using IVIS Spectrum imager (PerkinElmer). Minimum luminescence set at 500 units. (**M**) Kaplan–Meier survival plot for mouse experimental groups from (L) xenotransplanted with U87 cells carrying shScr (dashed black line) and *circACVR2A*(2–4) shRNA 1(red line) (*n* = 6 mice per group). *P*-value from log rank Mantel–Cox test.

Three shRNA constructs were designed to target the BSJ of *mmu_circAcvr2a*(2–4) and cloned into pLL3.7. This plasmid drives shRNA expression under the U6 promoter and, also, encodes CMV-driven GFP to tag transfected cells, *in vivo* (Supplementary Fig. S6C), and has been used previously to target a circRNA, *circFAT3*(1), by *in utero* electroporation (IUE) in neural progenitor cells of the dorsal cortex of the embryonic mouse brain [18]. These shRNAs silenced *mmu_circAcvr2a*(2–4) 36%–72%, with shRNA2 selected as it did not significantly silence the cognate *mmu_Acvr2a* mRNA (Fig. 6D).

IUE was performed at E14, and the positioning of the GFP-labelled, electroporated cells was determined after 3 days (Fig. 6E). Images of the dorsal cortex were binned into six sections from the subventricular zone to the marginal zone, representing the direction of neuronal cell migration from the inner to the outer region of the developing cortex (Fig. 6F). After 3 days, 51% of the scrambled shRNA-transfected cells (green fluorescence) had migrated to the marginal zone (Fig. 6G). Contrastingly, *mmu_circAcvr2a*(2–4)*-*deficient neurons showed significantly slower neuronal migration, with greater retention in the deeper layers and only 37% of GFP-positive cells in the marginal zone (Mann–Whitney, adjusted *P*-value = .012) (Fig. 6G). This mis-localization implicates a migration deficit, and/or an increased differentiation speed upon depletion of *circACVR2A*(2–4), in line with effects of silencing in hESCs.

### Silencing of *circACVR2A*(2–4) slows glioblastoma tumour progression, *in vivo*

GBM is an aggressive, heterogeneous brain tumour driven by a specialized subpopulation of treatment-resistant, self-renewing GSCs that originate from neural stem cells or progenitors [3]. As GSCs are responsible for tumour initiation, progression, and inevitable recurrence, treatments to drive their differentiation to improve survival, including the adaptation of retinoic acid therapy, continue to evolve [64].

Our previous data indicated that *circACVR2A*(2–4) is 2–8-fold more highly expressed in GBM tumours compared with matched non-malignant tissue from the same patient [65]. Silencing of *circACVR2A*(2–4) by shRNA was achieved in U87 human GBM cell lines (Fig. 6H), inducing neuronal differentiation phenotypes including increased number and length of neurites, increased SER Ridge and Edge scores, and reduced width:length and cell roundness scores as established by high-content phenotyping (Fig. 6I and J). Furthermore, transcriptomics profiling found that 22 of the top 30 enriched genesets in the silenced U87 lines were linked with neuronal differentiation (Fig. 6K and Supplementary Fig. S6D), mimicking the effects seen for *circACVR2A*(2–4) shRNA-silenced hESCs (Fig. 3E). Intracranial engraftment of the scrambled control and silenced U87 cell lines and bioluminescent tracking of tumours saw a slower growth rate for the silenced U87 cells (Fig. 6L). This translated into a significant increase in survival (*P* = .008), with a median increase of 40% (12 days) compared with scrambled control (Fig. 6M). This effect was confirmed for a second shRNA (Supplementary Fig. S6E), indicating that neuroectodermal differentiation may offer translational potential in treating GBM.

## Discussion

To date, RNAi-based circRNA knockdown screens have been restricted to non-pluripotent cell systems [66, 67]. By interrogating circRNA function across multiple hESC lineages, this study expands the current framework and highlights circRNAs as regulators of cell fate decisions rather than passive transcriptional by-products. In particular, *circACVR2A*(2–4) emerges as a determinant of pluripotency exit, positioning circRNAs within core developmental regulatory networks. This aligns with previous reports describing circRNAs as modulators of pluripotency and lineage commitment, such as *circBIRC6*, which regulates hESC identity through miRNA sequestration of miR-34/miR-145 [17]. The neuroectoderm (NE) phenotype following silencing of *circACVR2A*(2–4) is consistent with two, non-mutually exclusive models: where *circACVR2A*(2–4) directly specifies NE fate, or where *circACVR2A*(2–4) primarily sustains the pluripotent state until silenced, with neuroectoderm bias a known default state of pluripotency exit in the absence of alternative lineage cues [68]. While the neuronal differentiation/migration effect and attenuated GBM progression observed *in vivo* support the neuroectoderm specification role for *circACVR2A*(2–4) at this later stage of cell development, distinguishing between these models at the hESC stage remains to be conclusively demonstrated.

Exon–intron circRNAs, which predominate in the nucleus, have been shown to interact with U1 snRNA via RNA:RNA interaction [69], while long intronic circRNAs, such as *ci-ankrd52* have been shown to bind directly to elongating RNAPII and stimulate transcription of its cognate locus [70]. This study extends these findings by linking circRNA function directly to transcriptional control mechanisms governing differentiation [7]. When ascribing circRNA function, two often overlooked, yet critical, considerations are the stoichiometric ratio and localization of the proposed target. While the number of CAK complexes is not precisely known, all three components are within the lower third of the detectable proteome [71, 72]. Given that a 1:1 000 ratio of *circACVR2A*(2–4):CAK complexes used in the *in vitro* CAK assay impacted CTD phosphorylation, it is stoichiometrically plausible that the ∼52 *circACVR2A*(2–4) copies per hESC nucleus is sufficient to impact CAK activity and reduce RNAPII^S5p^ levels within the estimated 80 000 RNAPII holoenzymes per nucleus [73]. Certainly, the vast majority of lowly expressed, lineage-regulated circRNAs (less than three copies per cell) investigated herein, showed no spontaneous phenotypes following their knockdown. Contributing to the lack of phenotype for lowly expressed circRNAs could be an unfavourable stoichiometric ratio at the ES cell stage, therefore, these candidates may warrant further investigation through overexpression studies.

The substrate-selective nature of the interaction: where *circACVR2A*(2–4) potentiates phosphorylation of RNAPII-CTD but not CDK T-loop substrates, echoes the structural perturbations of CAK where MAT1 inclusion favours RNAPII phosphorylation over CDK phosphorylation by extending the interaction interface of CDK7 and CCNH [74, 75]. While experimentally validated under overexpression conditions, this interaction with *circACVR2A*(2–4) may be a critical scaffold contributing to substrate specific conformation of CAK for TFIIH-association as opposed to free CAK enzyme and T-loop phosphorylation [74, 76]. One limitation of this study is the lack of dissection of this precise interaction interface, which would demand protein and RNA structure-informed, targeted perturbations in a future study. Certainly, while targeted silencing of *circACVR2A*(2–4) reduces RNAPII^S5p^ by ∼70% without affecting cell cycle distribution and this is associated with NE differentiation phenotypically, the use of specific CDK7 inhibitors (samuraciclib, LDC4297) caused a partial differentiation, which may have been muted by simultaneously impacting both the RNAPII and CDK phosphorylation activities of the CAK complex. This is relevant in a clinical cancer setting [77] where further engineering of CDK inhibitors to decouple CAK complex activity may benefit patients with stem cell driven cancers. While the purified CAK assay demonstrates the capacity of *circACVR2A*(2–4) to influence CAK activity directly, the timing of the reduced RNAPII^S5p^ quantification (48 and 72 h post-knockdown), does not exclude potential earlier molecular differentiation, stress responses, or adhesion-related changes impacting RNAPII^S5p^ levels. Establishing causality will require parallel temporal analyses of RNAPII^S5p^ alongside pluripotency, early neural, and stress/adhesion markers.

Promoter-proximal pausing is a hallmark of pluripotent chromatin, maintaining developmental regulators in a poised but transcriptionally restrained state [77]. Genes specifying early ectoderm and NE fates exhibit some of the strongest RNAPII^S5p^ peaks downstream of the TSS [78]. As *circACVR2A*(2–4) silencing preferentially upregulated NE genes with the highest RNAPII^S5p^ enrichment at the TSS this aligns with established models in which loss of RNAPII^S5p^ destabilizes RNAPII pausing, prompting transcriptional activation of poised genes [79, 80]. The lineage selectivity observed here with *circACVR2A*(2–4) silencing more likely reflects the underlying distribution of paused polymerase across distinct developmental loci, rather than global transcriptional relaxation.

These findings have implications for disease contexts in which differentiation is disrupted, including cancer. The decreased *in vivo* neuronal migration seen here is consistent with mechanisms underlying cognitive, developmental, and psychiatric disorders collectively called neuronal migration disorders [81]. Excitingly, the suppression of GBM tumour growth by *circACVR2A* [2–4] targeting supports a link between enforced differentiation and reduced tumour proliferation. Given that cancer stem cells underly chemotherapy resistance, circRNAs that regulate differentiation states in these cells may represent tractable therapeutic targets [7, 82].

This work provides a molecular explanation for circRNA-mediated destabilization of RNAPII phosphorylation kinetics and release of poised genes as a possible mechanistic contributor to the loss of pluripotency and NE lineage commitment. Collectively, these findings establish *circACVR2A*(2–4) as an essential factor in restraining the differentiation of pluripotent stem cells and cancer stem cells, with the precise control of RNAPII initiation dynamics on poised transcriptional programs warranting further investigation. Given the silencing of this circRNA was able to slow progression of brain tumours by 40%, future research could exploit circRNAs as anticancer therapeutics.

## Supplementary Material

gkag964_Supplemental_Files

## Data Availability

RNA sequencing data have been deposited at Gene Expression Omnibus under accession number GSE245986. Mass spectrometry data have been submitted to ProteomeXchange via PRIDE with the accession PXD080807.

## Notes

Present address: Department of Genetics and Molecular Pathology, Centre for Cancer Biology, Adelaide University and SA Pathology, Adelaide, SA 5000, Australia

Present address: Centre for Cancer Biology, Adelaide University and SA Pathology, Adelaide, SA 5000, Australia
