# Supplementary material for: CircACVR2A(2–4) regulates stem cell pluripotency by uncoupling CDK-activating kinase functions
Source: Nucleic Acids Res. 2026 Oct 13;54(19):gkag964. doi: 10.1093/nar/gkag964 (PMC13659242; doi:10.1093/nar/gkag964)
Supplement: Supplementary file 1 [file gkag964_supplemental_files.zip › Webb et al SUPP NAR RESUB.pdf]

Supplemental information titles and legends

A

Cell Type	Replicate 1	Replicate 2	Replicate 3	Average	Common between replicates	Percent of Ave
H9 hESC	5328	5335	5835	5499.33	2215	40.28%
Definitive Endoderm	6677	7509	6694	6960	3019	43.38%
Floor Plate Precursors	11522	11507	13731	12253.3	6175	50.39%
Dopaminergic Neurons	13717	12910	7394	11340.3	3773	33.27%
Primitive Hepatocytes	5713	8530	7610	7284.33	3133	43.01%
Cardiomyocyte Spheres	8588	10187	7990	8921.67	4092	45.87%

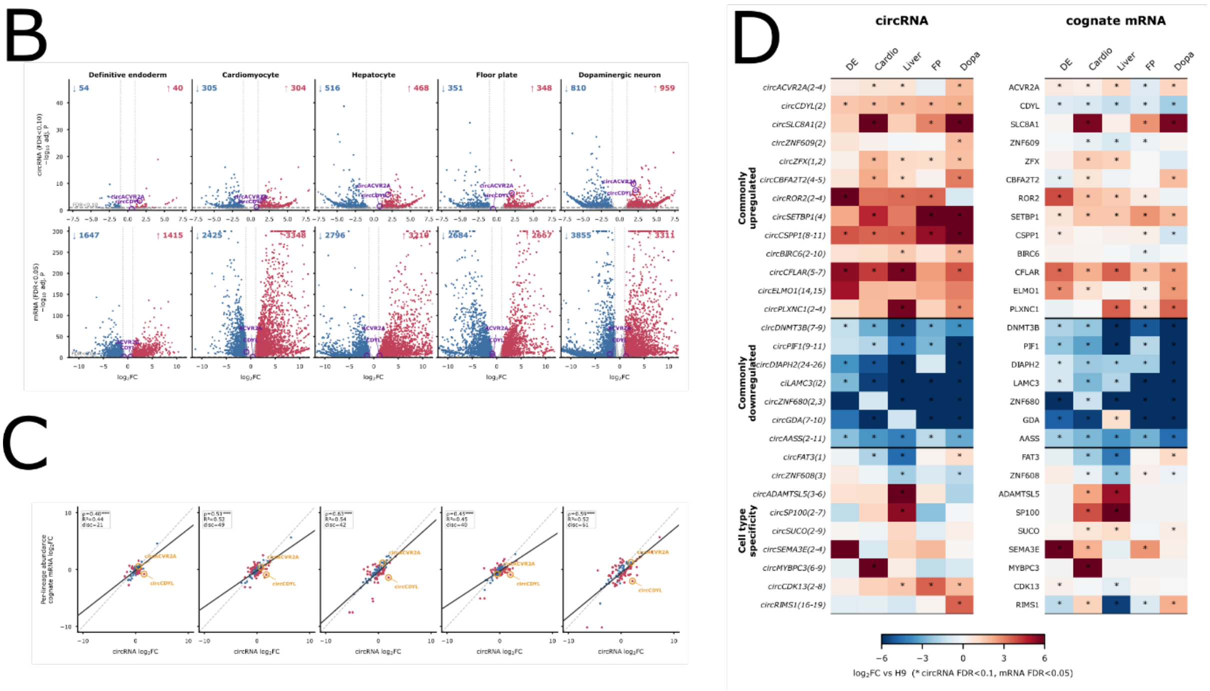

Figure S1:

(**A**) Number of unique and common circRNAs detected in each circRNA-seq library.

(**B**) Volcano plots of differential expression for circRNAs (top row, DESeq2) and mRNAs (bottom row, DESeq2), each lineage versus H9 hESCs. x, $\log_2$ fold change; y, $-\log_{10}$ Benjamini–Hochberg-adjusted P (capped at 300). Genes are called significant at $|\log_2 FC| \geq 1$ and FDR below a row-specific threshold: circRNA FDR < 0.10 (top) and mRNA FDR < 0.05 (bottom), the more permissive circRNA threshold offsetting the lower abundance and reduced DESeq2 power of circRNAs. Upregulated (red), downregulated (blue), and non-significant (grey) mRNAs and circRNAs; the

dashed line marks the FDR threshold and the arrows indicate significantly up- and down-regulated genes per panel. *circACVR2A*(2-4) and *circCDYL*(2) (top panels) and cognate mRNAs *ACVR2A* and *CDYL* (bottom panels) are ringed and labelled.

(**C**) Scatter plots of circRNA $\log_2$ fold change (DESeq2, each lineage vs H9 hESCs; x) against host-gene mRNA $\log_2$ fold change (y) for the 100 most abundant circRNAs, within each lineage; SynCRS spike-ins excluded. Discordance was tested per circRNA as $\Delta = \log_2 FC(\text{circRNA}) - \log_2 FC(\text{mRNA})$, $z = \Delta/\sqrt{(SE^2\text{circRNA} + SE^2\text{mRNA})}$, two-sided p, Benjamini–Hochberg FDR, with no significance prefilter (every circRNA with a finite $\log_2$FC and lfcSE tested). Significantly discordant circRNAs (red, FDR < 0.05), and concordant circRNAs (blue, FDR ≥ 0.05); named exemplars *circACVR2A*(2-4) and *circCDYL*(2) are shown in orange. Solid line, OLS fit; dashed line, y = x. Spearman's ρ and the coefficient of determination describe the overall correlation per panel. *** p<0.001, ** p<0.01, * p<0.05.

(**D**) Log2-fold change in circRNA (left panel) and cognate mRNA abundance (right panel) in the five differentiated cell lineages compared with H9 hESCs for the shortlisted candidate circRNAs. Grouped into circRNAs commonly upregulated and downregulated across differentiation and those that show cell-type specificity. Heat map shows log2-fold change in expression. Asterisk denotes significance (circRNA FDR < 0.1, mRNA FDR < 0.05).

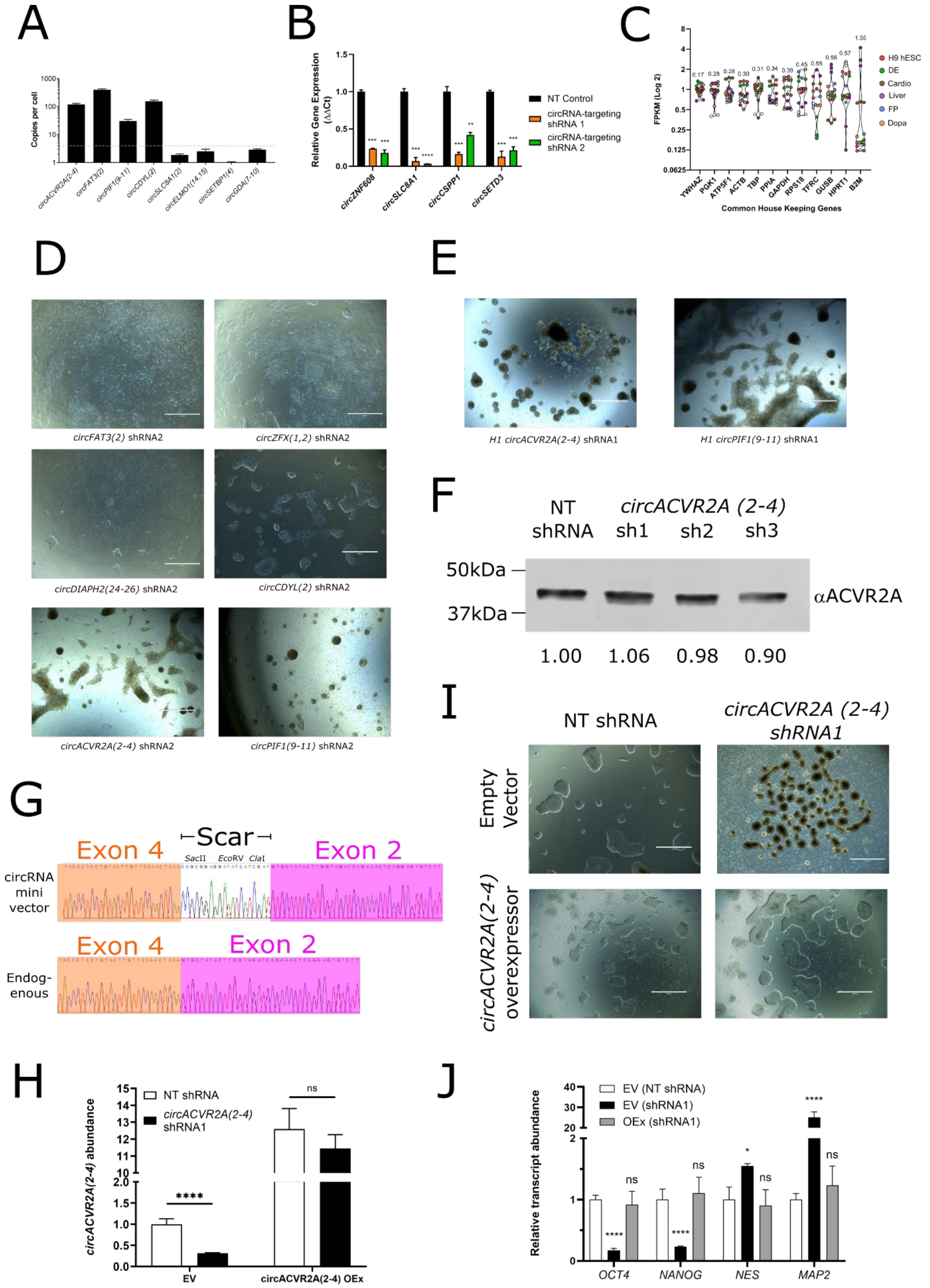
A
Copies per cell
circACVR2A(2-4)
circFAT3(2)
circPIF1(9-11)
circCDYL(2)
circSLC8A1(2)
circELMO1(14,15)
circSETBP1(4)
circGDA(7-10)
B
Relative Gene Expression (ΔΔCt)
NT Control
circRNA-targeting shRNA 1
circRNA-targeting shRNA 2
circZNF608
circSLC8A1
circCSPP1
circSETD3
C
FPKM (Log 2)
H9 hESC
DE
Cardio
Liver
FP
Dopa
Common House Keeping Genes
YWHAZ
PGK1
ATP5F1
ACTB
TBP
PPIA
GAPDH
RPS18
TFRC
GUSB
HPRT1
B2M
D
circFAT3(2) shRNA2
circZFX(1,2) shRNA2
circDIAPH2(24-26) shRNA2
circCDYL(2) shRNA2
circACVR2A(2-4) shRNA2
circPIF1(9-11) shRNA2
E
H1 circACVR2A(2-4) shRNA1
H1 circPIF1(9-11) shRNA1
F
NT shRNA
circACVR2A (2-4)
sh1
sh2
sh3
50kDa
37kDa
αACVR2A
1.00
1.06
0.98
0.90
G
Scar
Exon 4
Exon 2
SacII
EcoRV
ClaI
circRNA mini vector
Endog-enous
I
NT shRNA
circACVR2A (2-4) shRNA1
Empty Vector
circACVR2A(2-4) overexpressor
H
circACVR2A(2-4) abundance
NT shRNA
circACVR2A(2-4) shRNA1
ns
****
EV
circACVR2A(2-4) OEx
J
Relative transcript abundance
EV (NT shRNA)
EV (shRNA1)
OEx (shRNA1)
OCT4
NANOG
NES
MAP2

Figure S2:

(**A**) Absolute RT-qPCR on select circRNAs in hESCs. Data presented as circRNA copies per cell. Mean + SD (n=3 biological replicates).

(**B**) Relative expression of four lowly abundant circRNAs in hESC lines stably expressing either NT control shRNA, or each of two circRNA-specific shRNAs (shRNA1, shRNA2) as quantified by RT-qPCR. (n=3 biological replicates; Mean + SD)

(**C**) Expression variation among 12 common housekeeping transcripts shown as violin plots for FPKM (Log_2) during stem cell differentiation from mRNAseq data.

(**D**) Brightfield microscopy images of H9 hESC culture stably transduced with a second shRNA targeting one of six specific circRNAs after 7 days post selection in puromycin. 4x magnification, scale bar = 1,000 μm.

(**E**) Brightfield microscopy images of H1 hESC culture stably transduced with one shRNA targeting *circACVR2A(2-4)* (left panel) and *circPIF1(9-11)* (right panel) after 7 days post selection in puromycin. 4x magnification, scale bar = 1,000 μm.

(**F**) Western Blot for ACVR2A in hESCs stably expressing NT control or unique shRNAs targeting *circACVR2A(2-4)* (three shRNAs). Protein abundance indicated below each band based on intensity normalisation by total protein loading.

(**G**) Sanger sequencing of amplicon from circRNA mini vector overexpression of *circACVR2A*(2-4) (*top*) with molecular scar at BSJ comprising multiple cloning site restriction enzymes (*Sac*II, *Eco*RV and *Cla*I), compared to endogenous amplicon (*bottom*) showing *bona fide* BSJ.

(**H**) Relative abundance of *circACVR2A(2-4)* as assessed by RT-qPCR in empty vector (EV) or overexpression of *circACVR2A*(2-4) in circRNA mini vector with and without silencing by shRNA1 in H9 hESC line. Mean + SD; N = 3 replicates.

(**I**) Brightfield microscopy images of H9 hESC culture stably transduced with EV (*top*) or circRNA mini vector encoding *circACVR2A(2-4)* (*bottom*) and NT shRNA (*left*) or

shRNA1 targeting *circACVR2A(2-4)* (right panel) after 7 days post selection in puromycin. 4× magnification, scale bar = 1,000 µm.

(**J**) Relative abundance of representative pluripotency (*OCT4, NANOG*) and neuroectoderm (*NES, MAP2*) markers as assessed by RT-qPCR in empty vector (EV) or overexpression of *circACVR2A*(2-4) in circRNA mini vector (OEx) lines with silencing by NT shRNA or shRNA1 after 7 days. Mean + SD; N = 3 replicates.

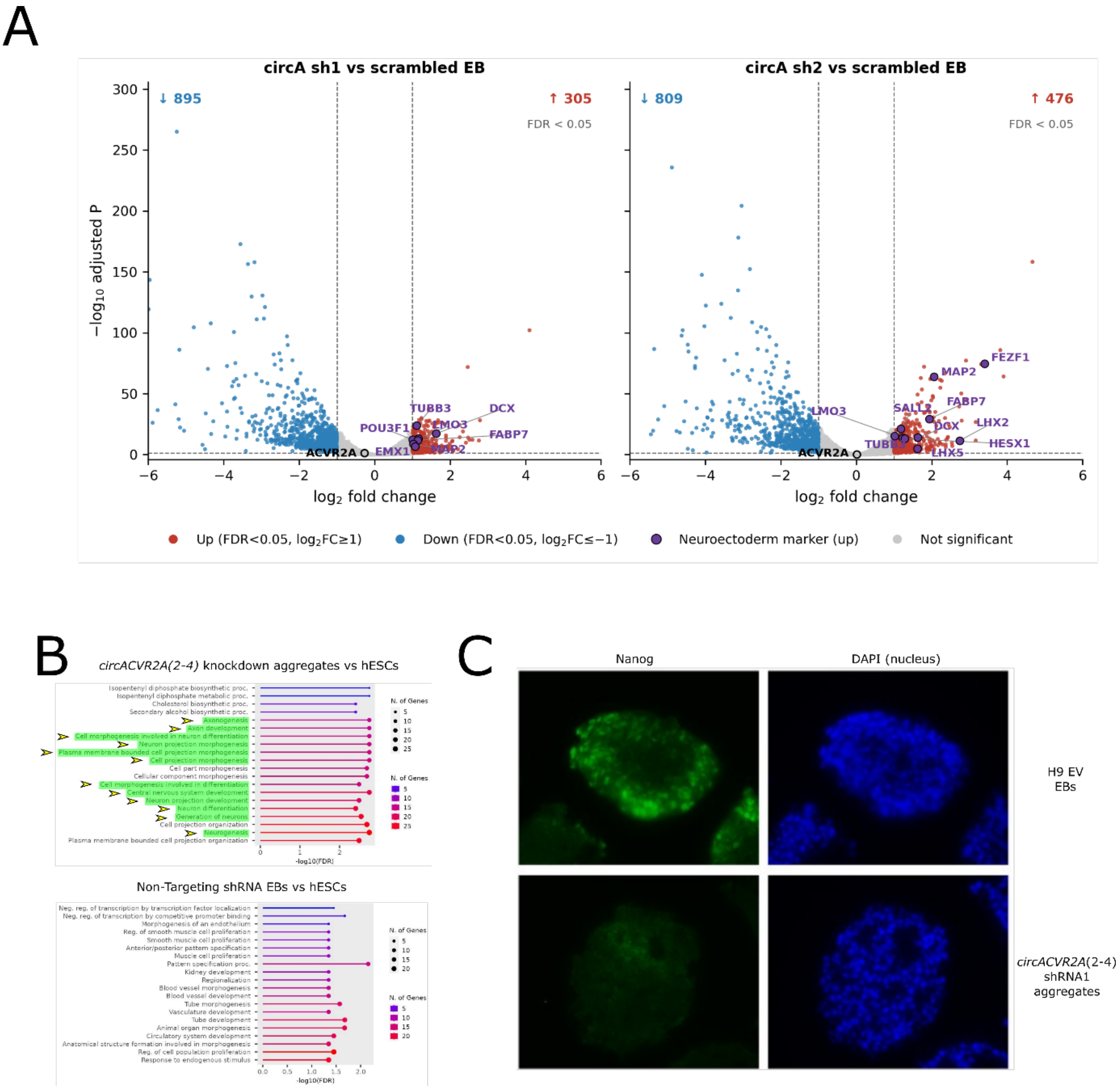

Figure S3:

(**A**) Volcano plot of mRNA differential expression (DESeq2) in *circACVR2A*(2-4)-knockdown H9 hESCs (shRNA1 and shRNA 2) versus scrambled-shRNA control EBs (three biological replicates per condition). x, $\log_2$ fold change (knockdown vs scrambled); y, $-\log_{10}$ Benjamini–Hochberg-adjusted P (capped at 300). Genes with FDR < 0.05 and $|\log_2 FC| \geq 1$ are coloured (up, red; down, blue); non-significant genes are grey; the dashed line marks the FDR threshold and the dotted lines $|\log_2 FC| = 1$. Neuroectoderm/neural-progenitor markers significantly upregulated (purple) are

labelled along with *ACVR2A* mRNA (open circle) and is not differentially expressed. (**B**) Geneset enrichment analysis from mRNA seq libraries from *circACVR2A(2-4)* knockdown aggregates (*top*) and non-targeting shRNA EBs (control, *bottom*) compared to adherent hESCs. Neuronal genesets highlighted in green (**C**) Immunofluorescence staining for NANOG (green) and DAPI (blue) in H9 EBs, either empty vector (pLKO.1) or *circACVR2A(2-4)* shRNA 1 aggregates. Images were taken at 10× magnification on Olympux AC70 using consistent exposure time for 488 filter.

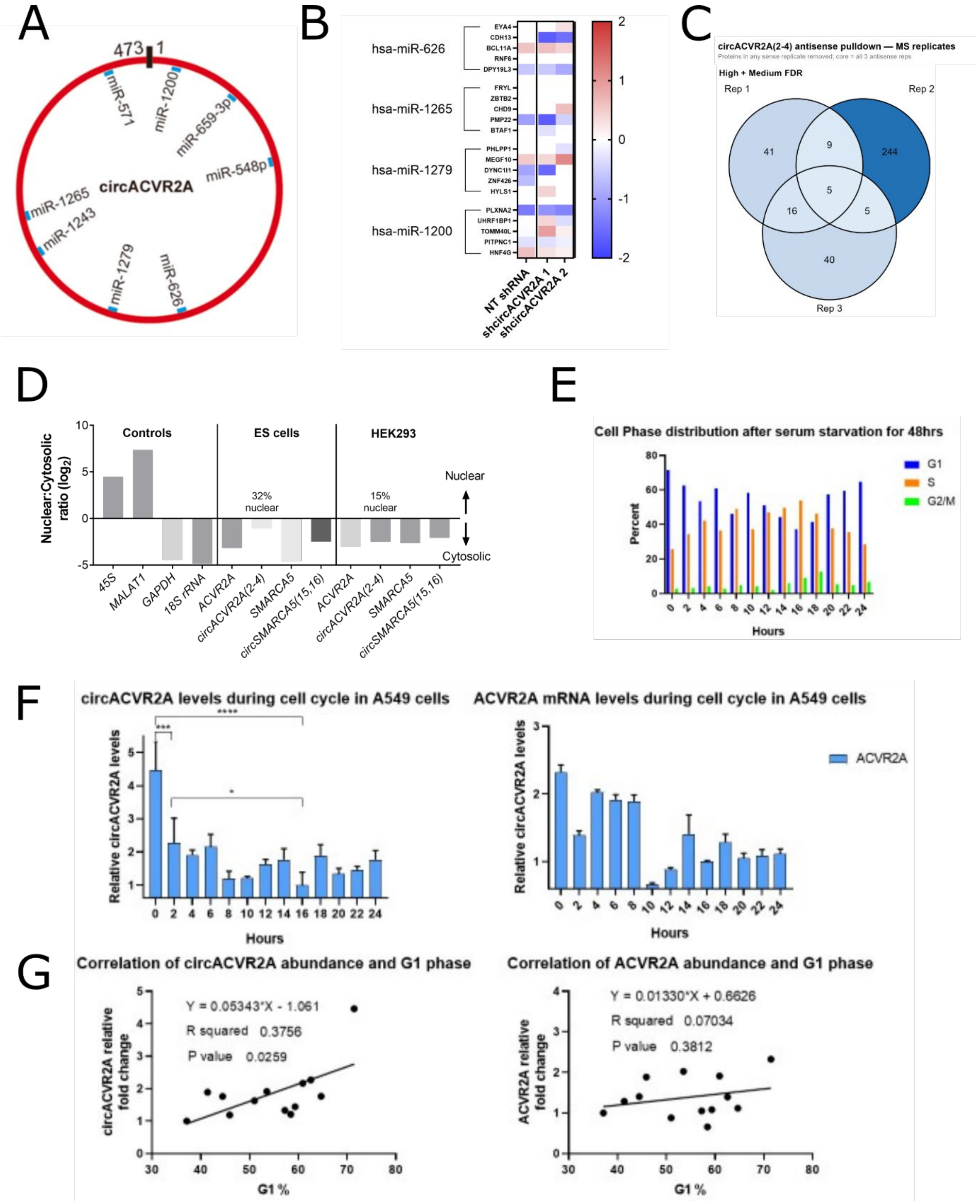

Supplemental Figure 4:

(**A**) Schematic of *circACVR2A(2-4)* highlighting predicted miRNA binding sites. (**B**) Heatmap showing fold change expression differences from mRNA-seq for downstream targets of predicted circACVR2A(2-4) miRNAs comparing EBs from NT

control shRNA or *circACVR2A(2-4)* shRNA 1 EBs, compared to adherent H9 hESCs. Grouped into known targets of each predicted miRNA based on miRbase predictions. No statistically significant differences in expression were found using correction for multiple comparisons using the Benjamini-Hochberg method; * FDR < 0.05, n = 3 replicates (**C**) Venn diagram showing medium and high FDR-enriched proteins compared to negative control identified in each of three mass spectrometry replicates of RIP-MS on *circACVR2A(2-4)*. (**D**) Nuclear:cytosolic fractionation RT-qPCR for circRNAs and mRNAs in H9 hESCs and HEK293T cells, with control nuclear (*45S rRNA*, *MALAT1*) and cytosolic RNAs (*18s rRNA* and *GAPDH*) as found in ES cells (**E**) Cell cycle distribution of A549 cells released from 48 hrs of serum starvation, flow cytometry analysis by propidium iodide staining at 2 hour intervals for 24 hours. Analysis by FloJo. Mean from n = 3 replicates (**F**) RT-qPCR on *circACVR2A(2-4)* and *ACVR2A* mRNA at 2 hourly timepoints as in (D). Data shown as Mean + SD (n = 3 biological replicates). (**G**) Scatter plots with regression line showing significant positive correlation between *circACVR2A(2-4)* expression, but not *ACVR2A* mRNA expression, and G1 occupancy.

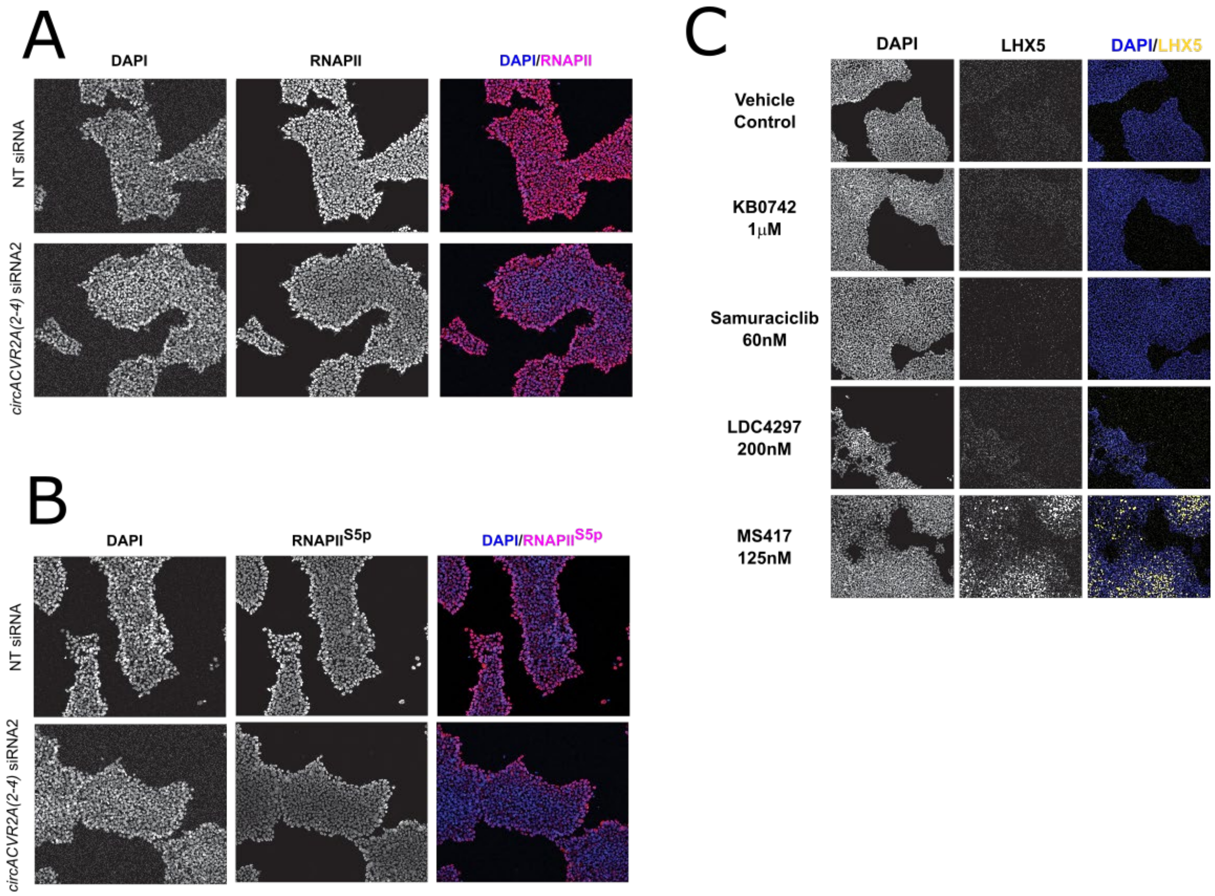

Supplemental Figure S5:

Fluorescence microscope images of H9 hESCs 72 hours after transfection with non-targeting (NT) siRNA and *circACVR2A(2-4)* siRNA #2 at 5 nM. Immunofluorescence for **(A)** RNAPII, or **(B)** RNAPII^{S5p} and DAPI to stain nuclei, with merged image showing signal overlap. Quantification shown in Figure 5D. (**C**) LHX5 immunofluorescence in H9 hESCs used to quantify NE differentiation induced by CDK7 (Samuraciclib, LDC4297), CDK9 (KB0742) and BET inhibitors (MS417).

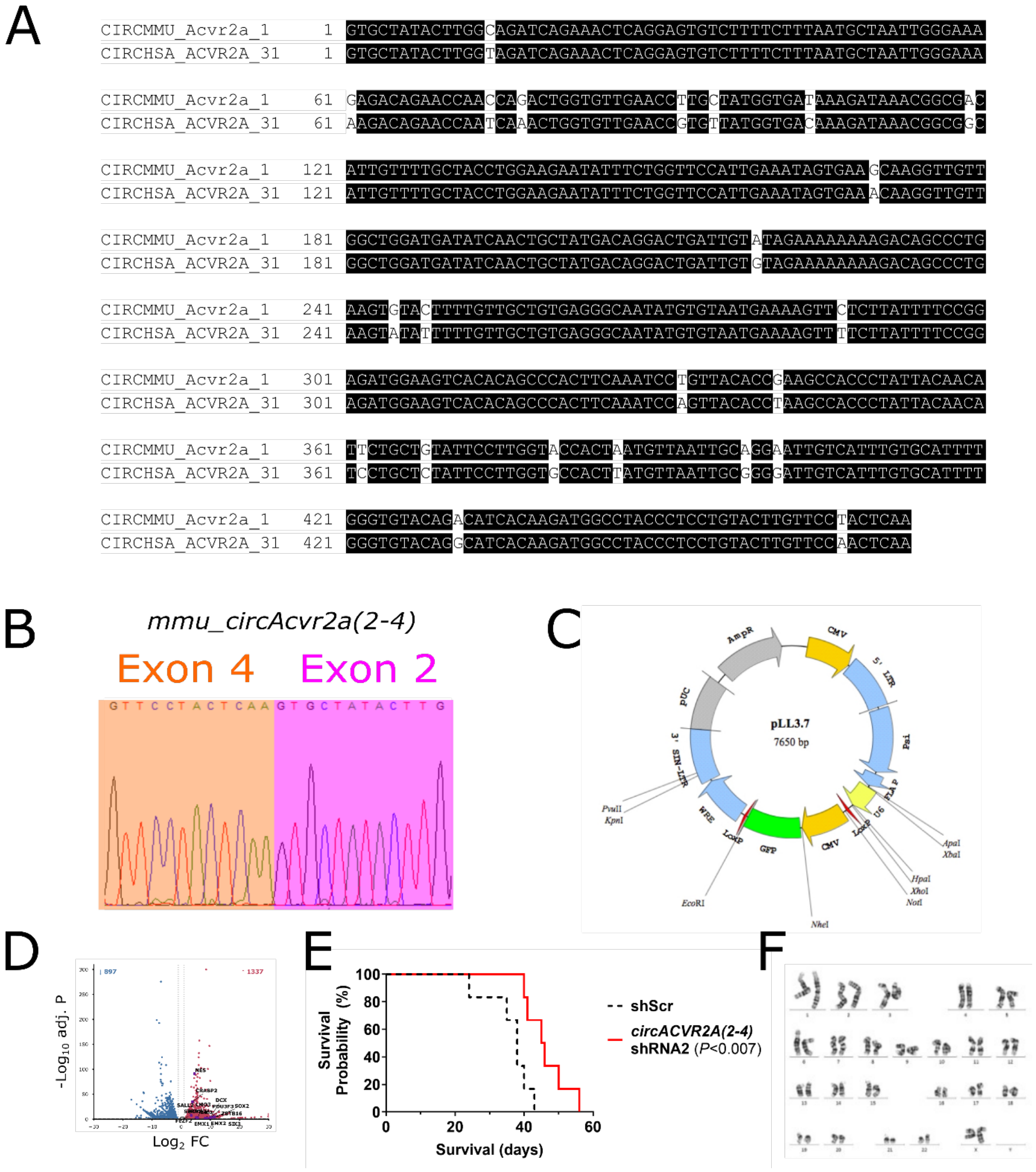

Supplemental Figure S6:

(**A**) CLUSTALX alignment of mouse (top) and human (bottom) *circACVR2A*(2-4) nucleotide sequences, showing matches (shaded) and mismatches (unshaded). (**B**) Sanger sequencing of PCR amplicon across BSJ of mouse *circAcvr2a*(2-4) showing BSJ junction.

(**C**) pLL3.7 shRNA expression construct (Addgene #11795) with shRNAs cloned into *Hpa*I/*Xho*I multiple cloning site and driven by U6 promoter for *in utero* electroporation experiments, which also produces GFP under CMV promoter. (**D**) Volcano plot of mRNA differential expression (DESeq2) in *circACVR2A*(2-4)-knockdown U87 cells versus scrambled-shRNA control (three biological replicates per condition). x, $\log_2$ fold change (scrambled control vs knockdown); y, $-\log_{10}$ Benjamini–Hochberg-adjusted P (capped at 300). Genes with FDR < 0.05 and $|\log_2 FC| \geq 1$ are coloured (up, red; down, blue); non-significant genes are grey; the dashed line marks the FDR threshold and the dotted lines $|\log_2 FC| = 1$. Neuroectoderm/neural-progenitor markers significantly upregulated (purple) are labelled along with *ACVR2A* mRNA (open circle) which is not differentially expressed ($\log_2 FC \approx 0.3$, adjusted P = 1). (**E**) Kaplan-Meier survival plot for mouse experimental groups xenotransplanted with U87 cells carrying shScr (dashed black line) and *circACVR2A(2-4)* shRNA 2 (red line) (n = 6 mice per group). p value from log rank Mantel-Cox test. (**F**) Karyotyping of H9 hESCs used in this study.
